# Endogenous Viral Element-Derived Piwi-Interacting RNAs (piRNAs) Are Not Required for Production of Ping-Pong-Dependent piRNAs from Diaphorina citri Densovirus

**DOI:** 10.1128/mBio.02209-20

**Published:** 2020-09-29

**Authors:** Jared C. Nigg, Yen-Wen Kuo, Bryce W. Falk

**Affiliations:** a Department of Plant Pathology, University of California, Davis, Davis, California, USA; McMaster University

**Keywords:** densovirus, *Diaphorina citri*, RNA interference, piRNA, small RNA

## Abstract

Piwi-interacting RNAs (piRNAs) are a class of small RNAs primarily responsible for silencing transposons in the animal germ line. The ping-pong cycle, the posttranscriptional silencing branch of the piRNA pathway, relies on piRNAs produced from endogenous transposon remnants to direct cleavage of transposon RNA via association with Piwi-family Argonaute proteins. In some mosquito species and mosquito-derived cell lines expressing a functionally expanded group of Piwi-family Argonaute proteins, both RNA and DNA viruses are targeted by piRNAs in a manner thought to involve direct processing of exogenous viral RNA into piRNAs. Whether viruses are targeted by piRNAs in nonmosquito species is unknown. Partial integrations of DNA and nonretroviral RNA virus genomes, termed endogenous viral elements (EVEs), are abundant in arthropod genomes and often produce piRNAs that are speculated to target cognate viruses through the ping-pong cycle. Here, we describe a Diaphorina citri densovirus (DcDV)-derived EVE in the genome of *Diaphorina citri*. We found that this EVE gives rise to DcDV-specific primary piRNAs and is unevenly distributed among *D. citri* populations. Unexpectedly, we found that DcDV is targeted by ping-pong-dependent virus-derived piRNAs (vpiRNAs) in *D. citri* lacking the DcDV-derived EVE, while four naturally infecting RNA viruses of *D. citri* are not targeted by vpiRNAs. Furthermore, a recombinant Cricket paralysis virus containing a portion of the DcDV genome corresponding to the DcDV-derived EVE was not targeted by vpiRNAs during infection in *D. citri* harboring the EVE. These results demonstrate that viruses can be targeted by piRNAs in a nonmosquito species independently of endogenous piRNAs.

## Abstract (precis)

Small RNAs serve as specificity determinants of antiviral responses in insects. Piwi-interacting RNAs (piRNAs) are a class of small RNAs found in animals, and their primary role is to direct antitransposon responses. These responses require endogenous piRNAs complementary to transposon RNA. Additionally, piRNAs have been shown to target RNA and DNA viruses in some mosquito species. In contrast to transposons, targeting of viruses by the piRNA pathway in these mosquito species does not require endogenous piRNAs. Here, we show that piRNAs target a DNA virus, but not RNA viruses, in an agricultural insect pest. We found that targeting of this DNA virus did not require endogenous piRNAs and that endogenous piRNAs did not mediate targeting of an RNA virus with which they shared complementary sequence. Our results highlight differences between mosquitoes and our experimental system and raise the possibility that DNA viruses may be targeted by piRNAs in other species.

## INTRODUCTION

The Piwi-interacting RNA (piRNA) pathway is a small RNA (sRNA)-guided gene silencing mechanism responsible for repressing transposable elements (TEs) in animals, and emerging evidence supports the idea of a role for this pathway in antiviral responses in some mosquito species and mosquito-derived cell lines (1–5). In Drosophila melanogaster, biogenesis of primary piRNAs begins with transcription of piRNA precursor transcripts from genomic regions called piRNA clusters (6). TE sequences integrated into piRNA clusters can become transcribed as part of a piRNA precursor transcript, and these precursor transcripts are processed into primary piRNAs that direct transcriptional and posttranscriptional silencing of TEs by association with the Piwi-family Argonaute proteins Piwi and Aubergine, respectively (1). During the ping-pong cycle, cleavage of sense TE RNA directed by Aubergine-bound antisense primary piRNAs triggers production of sense secondary piRNAs from cleaved TE RNA. Sense secondary piRNAs direct cleavage of antisense piRNA precursor transcripts via association with another Piwi-family Argonaute protein, Argonaute-3 (Ago3), to specifically amplify the response against active TEs (6). piRNAs are distinguished from other sRNAs by their size (24 to 32 nucleotides [nt]), nucleotide biases (uridine as the first nucleotide for primary piRNAs and adenine as the tenth nucleotide for secondary piRNAs, known as the 1U and 10A bias), and association with a Piwi-family Argonaute protein (1). Additionally, complementary piRNAs produced by the ping-pong cycle have 5′ ends separated by exactly 10 nt, known as the ping-pong signature (1).

The presence of ping-pong-dependent virus-derived piRNAs (vpiRNAs) during infection with several RNA viruses in *Aedes* and *Culex* mosquitoes and cell lines has been reported previously (2–5, 7–12), and reduced expression of piRNA pathway components leads to increased replication of Semliki Forest virus, Bunyamwera virus, Cache Valley virus, and Rift Valley fever virus in Aedes aegypti*-*derived Aag2 cells (2–5). Despite the prevalence of virus-derived piRNAs and evidence supporting their antiviral role, little is known regarding their biogenesis or antiviral function *in vivo*. *Ae. aegypti* expresses an expanded group of Piwi-family Argonaute proteins containing eight proteins (Piwi1 to Piwi7 and Ago3) rather than the three proteins seen in D. melanogaster (Piwi, Aub, and Ago3) (13). In Aag2 cells, Piwi5 and Ago3 are the primary Piwi-family Argonaute proteins required for biogenesis of vpiRNAs upon infection with Sindbis virus (9). Importantly, induction of the ping-pong cycle in mosquitoes is thought to involve biogenesis of primary vpiRNAs directly from viral RNA without the need for primary piRNAs derived from endogenous loci (9, 11). Thus, mosquitoes possess a unique piRNA pathway that produces vpiRNAs by virtue of novel functionality that has so far not been found in other groups of organisms. Indeed, the piRNA pathway does not play an antiviral role in D. melanogaster and ping-pong-dependent vpiRNAs have not been reported outside the mosquito lineage (14).

Arthropod genomes contain sequences derived from DNA viruses and nonretroviral RNA viruses (15–20). These sequences, termed endogenous viral elements (EVEs), are often enriched within piRNA clusters and serve as sources of primary piRNAs in many arthropod species (15–21). These observations have led to speculation that EVE-derived primary piRNAs may play an antiviral role by targeting cognate viruses in a manner analogous to piRNA-mediated repression of TEs via the ping-pong cycle (15–17). Importantly, such a role for EVE-derived piRNAs could theoretically be mediated by the canonical ping-pong cycle, potentially expanding the antiviral potential of the piRNA pathway beyond mosquitoes. In support of this idea, Whitfield et al. found that a single EVE-derived piRNA maps to the genome of Phasi Charoen-like virus (PCLV) in Aag2 cells, which are persistently infected with PCLV, and that a vpiRNA is produced from the complementary strand (16). Moreover, the authors found that knockdown of Piwi4 led to an ∼2-fold increase in the abundance of PCLV RNA in Aag2 cells (16). More recently, Tassetto et al. found that Piwi4 is required for vpiRNA maturation in Aag2 cells and binds specifically to piRNAs derived from EVEs (22). Furthermore, they found that insertion of EVE sequences into the 3′ untranslated region (UTR) of Sindbis virus reduced viral replication in a Piwi4-dependent manner in Aag2 cells (22). These results raise the possibility that EVE-derived piRNAs may target cognate viruses in these cells through an interaction with Piwi4; however, detailed sRNA profiles and more-thorough examinations of the interplay between virus-specific piRNAs derived from endogenous and exogenous sources are needed to establish the link between EVE-derived piRNAs and an antiviral response.

We previously identified EVEs present within the genomes of 48 arthropod species (15). Among the EVEs estimated to be similar enough to cognate exogenous viruses at the nucleotide level for piRNAs derived from them to potentially target viral RNA, Diaphorina citri densovirus (DcDV) and a DcDV-derived EVE located within the *Diaphorina citri* genome stood out as an EVE-virus pair sharing high nucleotide identity over a long region (15). Densoviruses are arthropod-infecting members of the family *Parvoviridae*. They have single-stranded DNA genomes, and the majority of currently known densoviruses cause highly pathogenic infections, although the existence of densoviruses causing infections with limited effects on host fitness is becoming increasingly recognized (23–26). We identified DcDV during a metagnomic screen of viruses associated with *D. citri* and subsequently found that the virus causes a persistent, maternally transmitted infection in *D. citri* (27). Also known as the Asian citrus psyllid, *D. citri* is a hemipteran insect pest and serves as a vector of *Candidatus* Liberibacter species that cause Huanglongbing, or citrus greening disease, in all types of commercially cultivated citrus (28). This disease represents the greatest threat to the citrus industry worldwide and has devastated many citrus-growing regions, such as in the U.S. state of Florida, where citrus production has decreased by 74% since the first report of citrus greening disease in the state in 2005 (29). Control strategies for the disease are limited and primarily involve removal of infected trees and management of *D. citri* populations with chemical insecticides (30). The continued spread of citrus greening disease and the development of insecticide resistance in field populations of *D. citri* highlight the need for new management approaches (30). Biological control strategies relying on infectious agents have proven to be effective methods for controlling insect pests, and the use of parasites and viruses has been proposed as possible vector control approaches for *D. citri* (31–34). Current understanding of immune processes in *D. citri* is limited, and studies in this field are needed to facilitate the development of effective biological control strategies. In particular, antiviral mechanisms in *D. citri* have not been experimentally evaluated. The presence of highly similar virus-EVE pairs, a lack of novel Piwi-family of Argonaute proteins like those observed in mosquitoes, and the need to study antiviral mechanisms in *D. citri* make this species an excellent and relevant model to study the interactions between EVE-derived piRNAs and corresponding viruses.

Here, we further characterized the DcDV-derived EVE present within the *D. citri* genome. We found that piRNAs are produced from this EVE in multiple tissue types and that this EVE is conserved among some, but not all, geographically distinct populations of *D. citri*. By analyzing sRNA profiles in *D. citri* insects lacking the EVE and infected with DcDV, we found that DcDV is targeted by ping-pong-dependent vpiRNAs independently of endogenous DcDV-specific piRNAs. Additionally, analysis of sRNA profiles during infection with several RNA viruses showed that they were not targeted by vpiRNAs in *D. citri*.

## RESULTS

### A DcDV-derived EVE is variably distributed in *D. citri* populations.

We previously identified a 621-bp DcDV-derived EVE located within a piRNA cluster on *D. citri* genomic scaffold 2850 by comparing deduced virus protein sequences to deduced *D. citri* genome-encoded protein sequences using BLASTx (15). This EVE was 85.5% identical to the corresponding region of the DcDV genome at the deduced amino acid level. To characterize this EVE at the nucleotide level, we aligned the nucleotide sequence of the DcDV genome to the region of the *D. citri* genome harboring these DcDV-derived EVEs. This was done using a draft of the *D. citri* genome assembled using PacBio long reads (Diaci2.0, ftp://ftp.citrusgreening.org/genomes/Diaphorina_citri/assembly/DIACI_v2.0/).

**FIG 1:**
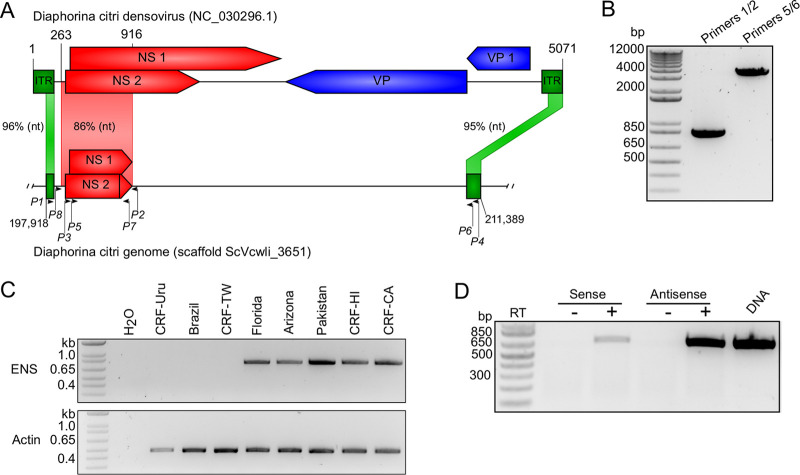
A DcDV-like EVE is present in the *D. citri* genome. (A) Organization of a DcDV-derived EVE identified in *D. citri* genomic scaffold ScVcwli_3651 by BLASTn followed by manual sequence alignment. The DcDV genome organization is shown on top with the corresponding region of scaffold ScVcwli_365 on the bottom. Numbers above and below the sequence depictions represent nucleotide positions. Blue and red shaded boxes with arrows represent open reading frames (ORFs), and the offset between boxes represents different reading frames. Green shaded boxes without arrows represent the ITRs. Vertical lines inside shaded boxes represent stop codons. The percentage of nucleotide identity between *D. citri* genomic and corresponding viral genomic regions is given. Annealing positions of PCR primers used in the experiments represented in panels B to D are shown with arrows. NS = nonstructural protein-encoding ORFs, VP = coat protein-encoding ORFs. (B) Confirmation of EVE presence by PCR using primers shown in panel A. The primer 5/6 PCR product was produced by nested PCR using as the template a 1:1,000 dilution of a PCR product produced with primers 3/4. (C) (Upper panel) PCR products produced using primers flanking ENS (primers 2 and 8). (Lower) PCR products produced using primers specific to *D. citri* actin (primers 9 and 10). (D) Primer 3 or primer 7 was used to generate cDNA from antisense or sense transcripts, respectively. cDNAs were used as the templates for PCR using primers 3 and 7. “+” and “-” indicate PCRs performed using cDNA prepared with (+) or without (-) reverse transcriptase (RT), respectively.

We found that this region of the *D. citri* genome contains sequences corresponding to the DcDV inverted terminal repeats (ITRs) and to two regions of the nonstructural protein (NS) gene cassette together spanning 862 bp. For simplicity and to distinguish different regions of this partial virus integration, we refer to these regions as endogenous ITRs (EITRs) and endogenous NS (ENS) (Fig. 1A). Notably, ENS, which spans nucleotides 197918 to 198700 within genomic scaffold ScVcwli_3651, shares 86% nucleotide identity with the corresponding region of the DcDV genome. To confirm the presence of these EVEs, we designed two sets of PCR primers to amplify this region from *D. citri* genomic DNA (diagrammed in Fig. 1A). Based on the sequence of genomic scaffold ScVcwli_3651 (Fig. 1A), the PCR product obtained with primers 1 and 2 was the anticipated size, but the PCR product produced with primers 5 and 6 was shorter than expected (∼13.4 kb expected versus ∼4 kb obtained) (Fig. 1B). This could have been due to misassembly of this region of the genome or could represent a polymorphism between the *D. citri* line used for genome sequencing and the *D. citri* line that we used for PCR. Sanger sequencing of these PCR products indicated that the sequences corresponding to DcDV were consistent with those indicated in the genome assembly (data not shown).

Our PCRs were performed using DNA extracted from *D. citri* insects collected from a colony located at the University of California Davis Contained Research Facility (CRF) that was started using insects collected in the U.S. state of California (this colony is designated CRF-CA). The *D. citri* reference genome was produced by sequencing DNA from insects collected in the U.S. state of Florida (35). Haplotype network analysis of global *D. citri* populations indicates the existence of 44 *D. citri* haplotypes belonging to two distinct lineages, denoted lineages A and B (36–38). The invasion history of *D. citri* out of Southern Asia has resulted in segregation of the two lineages, such that only lineage B is found in North America, while lineage A predominates in Southeast Asia, Africa, and South America (36). Besides CRF-CA, three other *D. citri* colonies are maintained at the CRF and these were started using *D. citri* insects collected in Taiwan, Uruguay, and the U.S. state of Hawaii (designated CRF-TW, CRF-Uru, and CRF-HI, respectively). To determine whether ENS is conserved among geographically distinct *D. citri* populations, we performed PCR using primers flanking ENS and DNA extracted from insects collected from CRF-CA, CRF-TW, CRF-Uru, and CRF-HI. We also included DNA extracted from field-collected *D. citri* insects from Pakistan, Brazil, and the U.S. states of Arizona and Florida. We obtained nearly identical PCR products from insects from CRF-CA, CRF-HI, Pakistan, Arizona, and Florida but obtained no PCR products from insects from CRF-TW, CRF-Uru, or Brazil (Fig. 1C; see also Fig. S1 in the supplemental material). Based on the distribution of the two *D. citri* lineages, these results suggest that ENS is absent in lineage A *D. citri*. Finally, strand-specific reverse transcription PCR (RT-PCR) results indicated that ENS was bidirectionally transcribed, although the majority of transcripts were antisense to the corresponding DcDV transcript (Fig. 1D).

10.1128/mBio.02209-20.1 FIG S1 Alignment of the sequences of the ENS PCR products shown in Fig. 1c. Sequences were aligned using ClustalW, and mismatched nucleotides are shaded. Download FIG S1, PDF file, 0.5 MB. Copyright © 2020 Nigg et al. 2020 Nigg et al. This content is distributed under the terms of the Creative Commons Attribution 4.0 International license.

### ENS and EITR give rise to DcDV-specific primary piRNAs.

**FIG 2:**
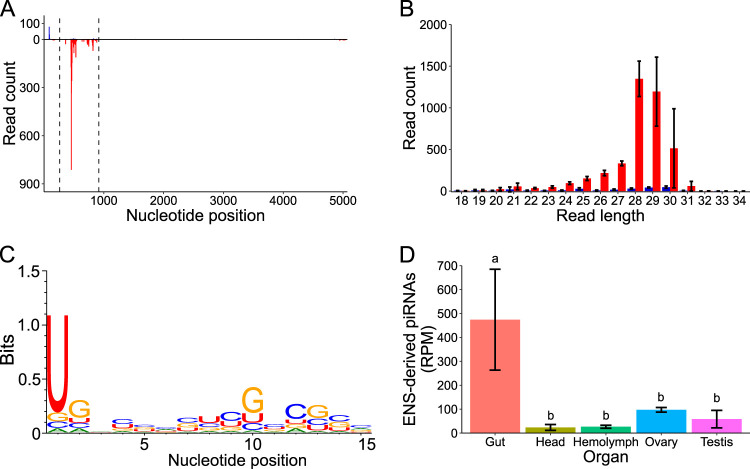
DcDV-specific piRNAs are produced from ENS in *D. citri* from CRF-CA. (A) Positions of all sRNAs from CRF-CA *D. citri* mapped to the DcDV genome. Dashed lines indicate the region of the DcDV genome corresponding to ENS. sRNAs are shown as mapped to the genomic strand containing the coding sequence for the NS proteins. Red = antisense sRNAs, blue = sense sRNAs. Read counts represent averages of results from three independent libraries. (B) Length distribution of sRNAs represented in panel A. Red = antisense, blue = sense. Read counts represent averages of results from three independent libraries. Error bars indicate standard deviations. (C) Sequence logo of sRNAs represented in panel A. Data represent results from three pooled libraries. (D) Abundance of 27-to-32-nt sRNAs from CRF-CA *D. citri* mapping to ENS in the indicated tissue types. Read counts for each library were normalized using the read counts per million mapped reads (RPM) method (59). Normalized read counts represent averages of results from three independent libraries. Error bars indicate standard deviations. Average RPM values for each tissue were compared by one-way analysis of variance (ANOVA) and Turkey’s honestly significant difference *post hoc* test. Significance is indicated by lowercase letters, and tissues sharing a letter do not have significantly different RPM values (*P* < 0.05).

To evaluate whether ENS and EITR give rise to virus-specific primary piRNAs, we mapped sRNAs from CRF-CA *D. citri* not infected with DcDV to the DcDV genome. We observed sRNAs mapping to the ITRs and to the negative strand within the portion of the DcDV genome corresponding to ENS (i.e., antisense to DcDV transcripts), but not sRNAs mapping to other regions (Fig. 2A). The size of these sRNAs was characteristic of piRNAs, and they possessed a 1U bias (Fig. 2B and C). Similar results were obtained for other *D. citri* populations that were not infected with DcDV and for which sRNA data sets are available (see Fig. S2A and B). As expected based on the lack of ENS in *D. citri* from Brazil or from CRF-Uru, DcDV-specific piRNAs were not observed in these insects (Fig. S2C and D). The DcDV ITRs are comprised of a hairpin present on both ends of the DcDV genome. Thus, it is not possible to determine whether the sRNAs mapping to EITR are specific to the positive or negative strand. For this reason, because of the small size of EITR, and because EITR does not correspond to a transcribed region of the DcDV genome (27), we chose to focus our analysis on piRNAs derived from ENS.

10.1128/mBio.02209-20.2 FIG S2 (A to D) (Left) Length distribution of all sRNAs from various *D. citri* populations mapped to the DcDV genome. Red = antisense, blue = sense. (Middle) Positions of all sRNAs from various *D. citri* populations mapped to the DcDV genome. sRNAs are shown as mapped to the genomic strand containing the coding sequence for the NS proteins. Red = antisense sRNAs, blue = sense sRNAs. (Right) Sequence logo of 27-to-32-nt sRNAs shown in the left and middle panels. sRNAs were purified from field-collected insects from China (GenBank accession no. SRX1164134) (A), the U.S. state of Florida (GenBank accession no. SRX1164135) (B), or Brazil (GenBank accession no. SRX1164127) (C) or from laboratory reared insects from CRF-Uru (D). Sequence data corresponding to panels A to C are described in reference 25 and reference 26. Download FIG S2, PDF file, 0.2 MB. Copyright © 2020 Nigg et al. 2020 Nigg et al. This content is distributed under the terms of the Creative Commons Attribution 4.0 International license.

piRNA expression patterns can display tissue specificity, and this could have important consequences for the ability of EVE-derived piRNAs to target cognate viruses. Thus, we sequenced sRNAs from dissected *D. citri* guts, heads, ovaries, testes, and hemolymph using insects collected from CRF-CA. DcDV-specific piRNAs derived from ENS were present in all tissues analyzed, but their expression level was significantly higher in *D. citri* guts than in any other tissue (Fig. 2D). The ping-pong cycle is restricted to germ line tissues in D. melanogaster (1); however, a comprehensive analysis of somatic sRNAs mapping to TEs genome-wide in 20 arthropod species indicated that somatic ping-pong amplification is widespread throughout arthropods despite having been independently lost in some species (39). To determine the tissues in which the ping-pong cycle is active in *D. citri*, we mapped sRNAs from CRF-CA *D. citri* guts, heads, hemolymph, ovaries, and testes to all TEs identified within the *D. citri* genome and analyzed the mapped 27-to-32-nt sRNAs for the presence of ping-pong signatures. We found evidence for ping-pong amplification of TE-derived piRNAs in all tissues examined (Fig. S3).

10.1128/mBio.02209-20.3 FIG S3 (A to E) Analysis of sRNAs from dissected CRF-CA *D. citri* tissues. Figures indicate mapping statistics for sRNAs mapping to all TEs identified in the *D. citri* genome. (A) sRNAs from three pools of 200 *D. citri* guts. (B) sRNAs from three pools of 200 *D. citri* heads. (C) sRNAs from three pools of hemolymph from 100 *D. citri* insects each. (D) sRNAs from three pools of 100 *D. citri* ovaries. (E) sRNAs from three pools of 100 *D. citri* testes. (Upper left panels) Length distribution of sRNAs mapping to TEs. Red = antisense, blue = sense. Read counts represent averages of results from three independent libraries. Error bars indicate standard deviations. (Upper right panels) Z-scores for the indicated overlap distances between the 5′ ends of complementary 27-to-32-nt sRNAs. Z-scores represent averages of results from three independent libraries. Errors bars indicate standard deviations. (Lower panels) Sequence logos for 27-to-32-nt sRNAs mapping to TEs. Lower left = antisense, lower right = sense. Data represent results from three pooled libraries. Download FIG S3, PDF file, 0.3 MB. Copyright © 2020 Nigg et al. 2020 Nigg et al. This content is distributed under the terms of the Creative Commons Attribution 4.0 International license.

### DcDV is targeted by ping-pong-dependent piRNAs independently of EVE-derived piRNAs.

**FIG 3:**
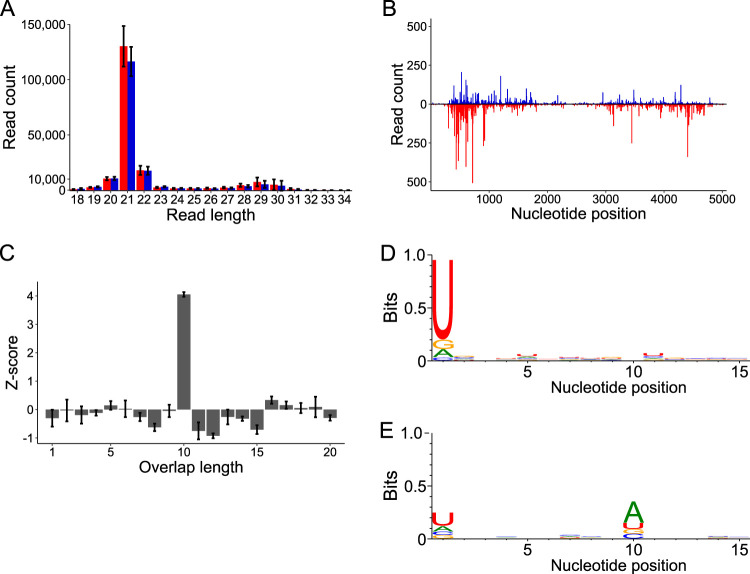
DcDV is targeted by ping-pong-dependent vpiRNAs in DcDV-infected *D. citri* insects from CRF-TW. (A) Length distribution of sRNAs mapping to transcribed regions of the DcDV genome in DcDV-infected *D. citri* insects from CRF-TW. To account for the bidirectional transcription strategy of DcDV, sRNA mapping polarity was assigned from mapping location based on the start and stop positions of the canonical DcDV transcripts. Red = antisense sRNAs, blue = sense sRNAs. Read counts represent averages of results from three independent libraries. Error bars indicate standard deviations. (B) Positions of 27-to-32-nt sRNAs represented in panel A. Red = antisense sRNAs, blue = sense sRNAs. Read counts represent averages of results from three independent libraries. (C) Z-scores for the indicated overlap distances between the 5′ ends of complementary 27-to-32-nt sRNAs represented in panel A. Z-scores represent averages of results from three independent libraries. Error bars indicate standard deviations. (D and E) Sequence logos for the 27-to-32-nt sRNAs represented in panel A. Sequence logos for antisense sRNAs (D) or sense sRNAs (E) are shown. Data represent results from three pooled libraries.

We previously found that *D. citri* from CRF-CA are resistant to infection with DcDV (27). In contrast, the virus is maintained as a persistent, maternally transmitted infection in *D. citri* from CRF-TW (27). Thus, to understand the sRNA-based response to DcDV infection in *D. citri*, we sequenced sRNAs from DcDV-infected *D. citri* from CRF-TW. These results revealed a major population of 21-nt sRNAs, indicating a small interfering RNA (siRNA)-based response (Fig. 3A). Unexpectedly, we also observed a smaller peak within the piRNA size range and we obtained 99.5% coverage of transcribed regions of the DcDV genome by mapping only 27-to-32-nt sRNAs (Fig. 3A and B). Complementary 27-to-32-nt sRNAs mapping to opposite strands throughout the DcDV genome possessed 5′ ends separated by 10 nt more often than expected by chance, an indication of ping-pong amplification (Z-score = 4.05 ± 0.08) (Fig. 3C). Moreover, we detected the 1U and 10A biases typical of ping-pong amplification in 27-to-32-nt sRNAs mapping antisense and sense to the canonical DcDV transcripts, respectively (Fig. 3D and E).

Results of PCR using primers flanking ENS suggested that ENS is not present in the genome of CRF-TW *D. citri* (Fig. 1C); thus, our observation of ping-pong-dependent DcDV-derived piRNAs in these insects suggests that DcDV is targeted by piRNAs independently of ENS. However, it is possible that ENS is present in CRF-TW *D. citri*, but resides in different genomic context than that seen in other populations. ENS is not identical to the corresponding region of the DcDV genome (Fig. 1C). Thus, some piRNAs derived from ENS do not perfectly map to DcDV, providing a means by which to distinguish some ENS-derived piRNAs from DcDV-derived piRNAs. To rule out the possibility that ENS is present in the genome of CRF-TW *D. citri*, we mapped 27-to-32-nt sRNAs from CRF-TW *D. citri* to the DcDV genome without allowing any mismatches. When the unmapped reads from this analysis were mapped to ENS without allowing any mismatches, no reads mapped. In contrast, we obtained an average of 55.8% coverage of the ENS sequence when the same analysis was performed using sRNAs from CRF-CA *D. citri* (data not shown). This result indicates that ENS is indeed not present in the genome of CRF-TW *D. citri* and that the targeting of DcDV by ping-pong-dependent vpiRNAs in these insects is independent of ENS-derived piRNAs.

We cannot exclude the possibility that the genome of CRF-TW *D. citri* harbors a different piRNA-producing DcDV-derived EVE. Because DcDV is maternally transmitted to 100% of the progeny of CRF-TW females (27), it is not possible to determine the repertoire of endogenous piRNAs in these insects in the absence of DcDV infection. Thus, we analyzed the sRNAs present in *D. citri* from CRF-Uru, as these insects lack ENS and are not infected with DcDV (Fig. 1C). We found that no DcDV-specific piRNAs were produced in uninfected CRF-Uru *D. citri*, indicating that these insects do not harbor a piRNA-producing DcDV-derived EVE (Fig. S2D). The absence of DcDV-specific piRNAs was not due to a lack of piRNAs in general, as we detected abundant ping-pong-dependent TE-derived piRNAs in these insects (Fig. S4). In contrast to *D. citri* from CRF-CA, which are resistant to DcDV infection (27), we found that *D. citri* from CRF-Uru were susceptible to DcDV infection by both oral acquisition and intrathoracic injection (Fig. S5A and B). Moreover, the progeny of CRF-Uru *D. citri* infected with DcDV were also infected with the virus (Fig. S5C). Analysis of sRNAs from the progeny of CRF-Uru *D. citri* that had been infected with DcDV by intrathoracic injection revealed the presence of ping-pong-dependent DcDV-derived piRNAs (ping-pong Z-score = 2.55) (Fig. S5D to H). Together, these results demonstrate that DcDV is targeted by ping-pong-dependent vpiRNAs in two populations of *D. citri* independently of EVE-derived piRNAs.

10.1128/mBio.02209-20.4 FIG S4 (A to E) Analysis of sRNAs from CRF-Uru *D. citri*. Figures indicate mapping statistics for sRNAs mapping to all TEs identified in the *D. citri* genome. (A) Length distribution of sRNAs mapping to TEs. Red = antisense, blue = sense. (B) Z-scores for the indicated overlap distances between the 5′ ends of complementary 27-to-32-nt sRNAs. (C and D) Sequence logos for 27-to-32-nt sRNAs mapping to TEs. (C) Antisense sRNAs. (D) Sense sRNAs. Download FIG S4, PDF file, 0.1 MB. Copyright © 2020 Nigg et al. 2020 Nigg et al. This content is distributed under the terms of the Creative Commons Attribution 4.0 International license.

10.1128/mBio.02209-20.5 FIG S5 (A) Titer of DcDV following intrathoracic injection of virions into uninfected *D. citri* insects from CRF-Uru. Each data point represents three pooled insects (*n* = 5). The curve was smoothed using locally estimated scatterplot smoothing (LOESS) regression. (B) Titer of DcDV following feeding of virions to uninfected *D. citri* insects from CRF-Uru. Virions present in a 15% sucrose solution were acquired by *D. citri* for 96 h by membrane feeding. After the feeding period, insects were moved to *Citrus macrophylla* plants; this represents day 0 postfeeding. Each data point represents three pooled insects (*n* = 5). The curve was smoothed using LOESS regression. (C) Titer of DcDV in F1 progeny of the insects represented in panel A (designated by injection) and panel B (designated by feeding). *n* = 10 individual insects. Individual insects were collected on the day of emergence to adulthood. (D to H) DcDV is targeted by ping-pong-dependent vpiRNAs in DcDV-infected *D. citri* insects from CRF-Uru. sRNAs were purified from the progeny of CRF-Uru *D. citri* that had been infected with DcDV by intrathoracic injection. (D) Length distribution of sRNAs mapping to transcribed regions of the DcDV genome in DcDV-infected *D. citri* insects from CRF-Uru. To account for the bidirectional transcription strategy of DcDV, sRNA mapping polarity was assigned from the mapping location based on the start and stop positions of the canonical DcDV transcripts. Red = antisense sRNAs, blue = sense sRNAs. (E) Positions of 27-to-32-nt sRNAs represented in panel D. Red = antisense sRNAs, blue = sense sRNAs. (F) Z-scores for the indicated overlap distances between the 5′ ends of complementary 27-to-32-nt sRNAs represented in panel D. (G and H) Sequence logos for the 27-to-32-nt sRNAs represented in panel D. Sequence logos for antisense sRNAs (G) or sense sRNAs (H) are shown. Download FIG S5, PDF file, 0.2 MB. Copyright © 2020 Nigg et al. 2020 Nigg et al. This content is distributed under the terms of the Creative Commons Attribution 4.0 International license.

### RNA viruses of *D. citri* are not targeted by piRNAs.

Besides DcDV, there are five other viruses known to infect *D. citri*: Diaphorina citri reovirus, Diaphorina citri picorna-like virus, Diaphorina citri bunyavirus, Diaphorina citri-associated c virus, and Diaphorina citri flavi-like virus (34, 40–42). To determine whether piRNAs represent part of a general response to viruses in *D. citri*, we mapped sRNAs from publicly available sRNA libraries known to be derived from insects infected with one or more of each of these viruses to the corresponding viral genomes (with the exception of Diaphorina citri-associated c virus, for which no such sRNA library exists) (34, 40). We detected a prominent peak at 21 nt for each virus, indicating a siRNA-based response (Fig. S6). While virus-derived sRNAs within the piRNA size range were present during infection with all four viruses, there were no peaks above background levels within the piRNA size range and 27-to-32-nt reads lacked signatures typical of primary or ping-pong-dependent piRNAs (Fig. S6). These results indicate that viruses in general are not targeted by piRNAs in *D. citri*.

10.1128/mBio.02209-20.6 FIG S6 (A to E) Analysis of sRNAs from various field-collected *D. citri* populations infected with one or more *D. citri*-specific viruses. (A) *D. citri* from the U.S. state of Florida infected with Diaphorina citri flavi-like virus (sRNA GenBank accession no. SRX1164135, viral genome GenBank accession no. NC_030453.1). (B) *D. citri* from China infected with Diaphorina citri reovirus (sRNA GenBank accession no. SRX1164134, viral genome GenBank accession no. KT698830.1, KT698831.1, KT698832.1, KT698833.1, KT698834.1, and KT698836.1). (C) *D. citri* from China infected with Diaphorina citri picorna-like virus (sRNA GenBank accession no. SRX1164134, viral genome GenBank accession no. KT698837.1). (D) *D. citri* from China infected with Diaphorina citri bunyavirus (sRNA GenBank accession no. SRX1164134, viral genome GenBank accession no. KT698825.1, KT698824.1, KT698823.1). (E) *D. citri* from Brazil infected with Diaphorina citri picorna-like virus (sRNA GenBank accession no. SRX1164127, viral genome GenBank accession no. KT698837.1). (Upper left panels) Length distribution of sRNAs mapped to the various viral genomes. Red = antisense, blue = sense. (Upper right panels) Z-scores for the indicated overlap distances between the 5′ ends of complementary 27-to-32-nt sRNAs mapping to opposite strands of the various viral genomes. Sequence data are described in reference 25 and reference 26. (Lower panels) Sequence logos for 27-to-32-nt sRNAs mapping to the various viral genomes. Lower left = antisense, Lower right = sense. Download FIG S6, PDF file, 0.3 MB. Copyright © 2020 Nigg et al. 2020 Nigg et al. This content is distributed under the terms of the Creative Commons Attribution 4.0 International license.

### A recombinant reporter virus harboring DcDV sequence is not targeted by ENS-derived piRNAs in *D. citri*.

As has been observed for RNA viruses in mosquitoes, our results suggest that DcDV is targeted by ping-pong-dependent vpiRNAs due to the *de novo* production of vpiRNAs from exogenous DcDV RNA. If EVE-derived piRNAs were to prime the ping-pong cycle in this context, it would be difficult to distinguish priming driven by EVE-derived piRNAs from priming driven by virus-derived vpiRNAs. Because our results suggest that RNA viruses in general are not targeted by vpiRNAs in *D. citri*, we sought to construct a recombinant RNA virus harboring EVE sequence in order to study potential priming of the ping-pong cycle by EVE-derived piRNAs without the background of vpiRNAs produced directly from viral RNA. For this purpose, we used Cricket paralysis virus (CrPV), a dicistrovirus that was originally isolated from field crickets (43). Due to the broad experimental host range of CrPV, including *D. citri* (E. Matsumura, unpublished data), and the availability of an infectious clone, CrPV is often used to study antiviral mechanisms in insects (44, 45).

**FIG 4:**
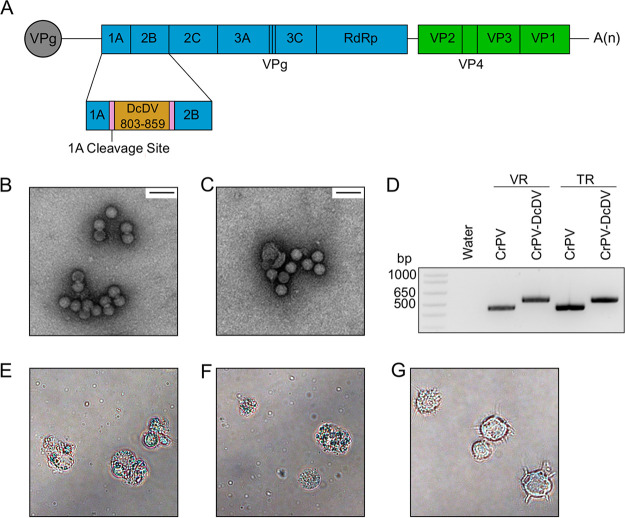
Construction of CrPV-DcDV, a recombinant CrPV mutant containing 57 nt of sequence from the DcDV genome. (A) Genome organization of CrPV-DcDV. Blue rectangles represent the CrPV nonstructural proteins. RdRp = RNA-dependent RNA polymerase. VPg = viral protein genome-linked. Green boxes represent the CrPV structural proteins. The orange rectangle represents the recombinant DcDV sequence, which corresponds to nucleotides 803 to 859 from the DcDV genome. Pink rectangles represent the cleavage site at which the 1A protein is released from the polyprotein. (B and C) Electron micrographs of wild-type CrPV (B) or CrPV-DcDV (C) virions purified from S2 cells transfected with viral RNA (×50,000 magnification). Scale bar is 50 nm. (D) RT-PCR products produced using primers flanking the site into which recombinant DcDV sequence was inserted in CrPV-DcDV (primers 11 and 12). RNA extracted from purified virions (VR) or *in vitro*-transcribed viral RNA (TR) was used as a template. (E to G) Bright-field microscopy images of S2 cells infected with wild-type CrPV virions (E) or CrPV-DcDV virions (F) or mock infected (G). Images were acquired 72 h post-infection.

To directly test the hypothesis that EVE-derived primary piRNAs can prime ping-pong amplification during infection with viruses sharing complementary sequence, we inserted 57 nt from the DcDV genome (from a region corresponding to ENS) into the CrPV genome between the 1A and 2B coding sequences flanked by duplicate copies of the 1A cleavage site (Fig. 4A; see also Fig. S7). The recombinant DcDV sequence was inserted into the CrPV genome such that ENS-derived primary piRNAs were antisense to the viral coding sequence and, in analogy to the mechanisms of ping-pong amplification in the context of TEs, should theoretically have been capable of directing cleavage of viral mRNAs. This recombinant virus was designated CrPV-DcDV. While the 57-nt inserted region was relatively small, piRNA target sites corresponding to single piRNAs are sufficient for induction of ping-pong amplification in D. melanogaster and a single piRNA produced from a PCLV-derived EVE was previously proposed to mediate a piRNA-based antiviral response in Aag2 cells (16, 46–48). Additional analysis of the sRNA mapping data from DcDV-uninfected CRF-CA *D. citri* shown in Fig. 2A indicated that this 57-nt region gave rise to an average of 171.7 27-to-32-nt sRNAs made up of an average of 30.7 unique sequences in the three sRNA libraries analyzed (data not shown).

10.1128/mBio.02209-20.7 FIG S7 Alignment of a portion of the CrPV-DcDV genome, the recombinant DcDV sequence present within CrPV-DcDV, the region of ENS corresponding to the recombinant DcDV sequence present within CrPV-DcDV (represented by *D. citri* genomic scaffold 2850 GenBank accession no. NW_007380266), and a portion of the wild-type CrPV sequence. Numbers in parentheses indicate the nucleotide positions of the sequence shown. The CrPV 1A cleavage site is shown in lowercase letters. The recombinant DcDV sequence present within CrPV-DcDV and the corresponding region of ENS are shown in italics. Download FIG S7, PDF file, 0.02 MB. Copyright © 2020 Nigg et al. 2020 Nigg et al. This content is distributed under the terms of the Creative Commons Attribution 4.0 International license.

Virions produced following transfection of S2 cells with *in vitro*-transcribed CrPV-DcDV RNA were indistinguishable from those produced following transfection with wild-type CrPV RNA (Fig. 4B and C), and RT-PCR of RNA purified from CrPV-DcDV virions indicated that the recombinant sequence was retained (Fig. 4d). Furthermore, infection of S2 cells with either wild-type CrPV or CrPV-DcDV virions resulted in the cytopathic effects characteristic of CrPV infection (Fig. 4E and F).

**FIG 5:**
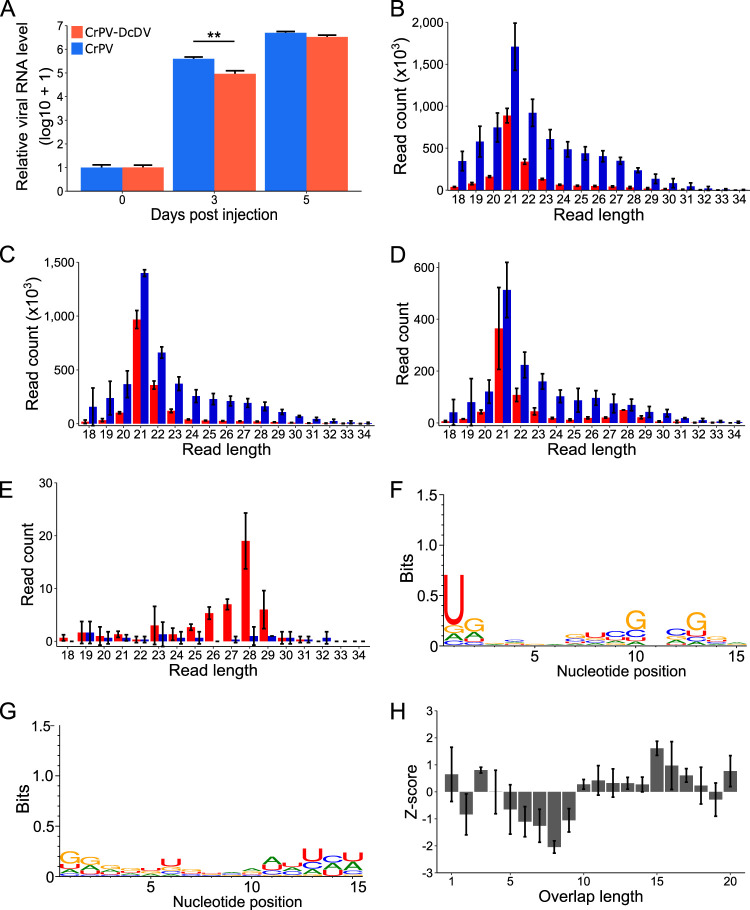
CrPV-DcDV is not targeted by piRNAs during infection initiated by intrathoracic injection of purified virions. (A) Relative viral RNA levels during infection with wild-type CrPV or CrPV-DcDV. Infection was initiated by intrathoracic injection of CRF-CA *D. citri* with 1,000 TCID_50_ units of purified virions per insect. Viral RNA levels were assessed by RT-qPCR and normalized based on the expression of actin. The amount of viral RNA present on day 0 was set as 1, and log_10_ + 1 levels of viral RNA are shown relative to this value. Bars represent the average viral RNA level in five pools of three insects. Error bars indicate standard errors of the means. ** = *P* < 0.01, two-tailed T-test. (B to F) Analysis of sRNA sequencing data of sRNAs purified from CRF-CA *D. citri* infected with wild-type CrPV or CrPV-DcDV by intrathoracic injection as described for panel A. sRNA was purified from pools of 25 *D. citri* collected 5 days postinjection. (B) Length distribution of sRNAs from wild-type CrPV-infected *D. citri* mapped to the wild-type CrPV genome. Red = antisense, blue = sense. Read counts represent averages of results from three independent libraries. Error bars indicate standard deviations. (C) Length distribution of sRNAs from CrPV-DcDV-infected *D. citri* mapped to the CrPV-DcDV genome. Red = antisense, blue = sense. Read counts represent averages of results from three independent libraries. Error bars indicate standard deviations. (D) Length distribution of sRNAs from CrPV-DcDV-infected *D. citri* mapped to the recombinant DcDV sequence present within the CrPV-DcDV genome. Red = antisense, blue = sense. Read counts represent averages of results from three independent libraries. Error bars indicate standard deviations. (E) Length distribution of sRNAs from wild-type CrPV-infected *D. citri* mapped to the recombinant DcDV sequence present within the CrPV-DcDV genome. Red = antisense, blue = sense. Read counts represent averages of results from three independent libraries. Error bars indicate standard deviations. (F and G) Sequence logos for 27-to-32-nt sRNAs from CrPV-DcDV-infected *D. citri* mapped to the recombinant DcDV sequence present within the CrPV-DcDV genome. Sequence logos for antisense sRNAs (F) or sense sRNAs (G) are shown. Data represent results from three pooled libraries. (H) Probability of overlap of the 5′ ends of complementary 27-to-32-nt sRNAs mapping to opposite strands of the recombinant DcDV sequence present within the CrPV-DcDV genome during infection with CrPV-DcDV. Probabilities are shown for the indicated overlap distances and represent averages of results from three independent libraries. Error bars indicate standard deviations. The average Z-score and the standard deviation of the Z-score for an overlap length of 10 nt are shown.

To determine whether ENS-derived piRNAs mediate a response to CrPV-DcDV, we inoculated CRF-CA *D. citri* with 1,000 50% tissue culture infectious dose (TCID_50_) units of wild-type CrPV or CrPV-DcDV per insect by intrathoracic injection. We observed a significant difference in viral RNA levels for wild-type CrPV and CrPV-DcDV 3 days post-injection (Fig. 5A). By 5 days postinjection, CrPV-DcDV RNA levels remained lower than those of wild-type CrPV RNA, but the difference was not statistically significant (Fig. 5A). RT-PCR results indicated the recombinant DcDV sequence was retained throughout the course of infection with CrPV-DcDV (Fig. S8). To determine if CrPV-DcDV was targeted by piRNAs, we sequenced sRNAs from insects collected 5 days post-injection. We observed a siRNA-based response targeting the entirety of both viral genomes, including the recombinant DcDV sequence within the CrPV-DcDV genome (Fig. 5B to D; see also Fig. S9A and B). When sRNAs from *D. citri* infected with wild-type CrPV were mapped to the same DcDV-derived sequence, a peak within the piRNA-size range was observed due to the production of piRNAs from ENS, but no 21-nt peak was seen (Fig. 5E).

**FIG 6:**
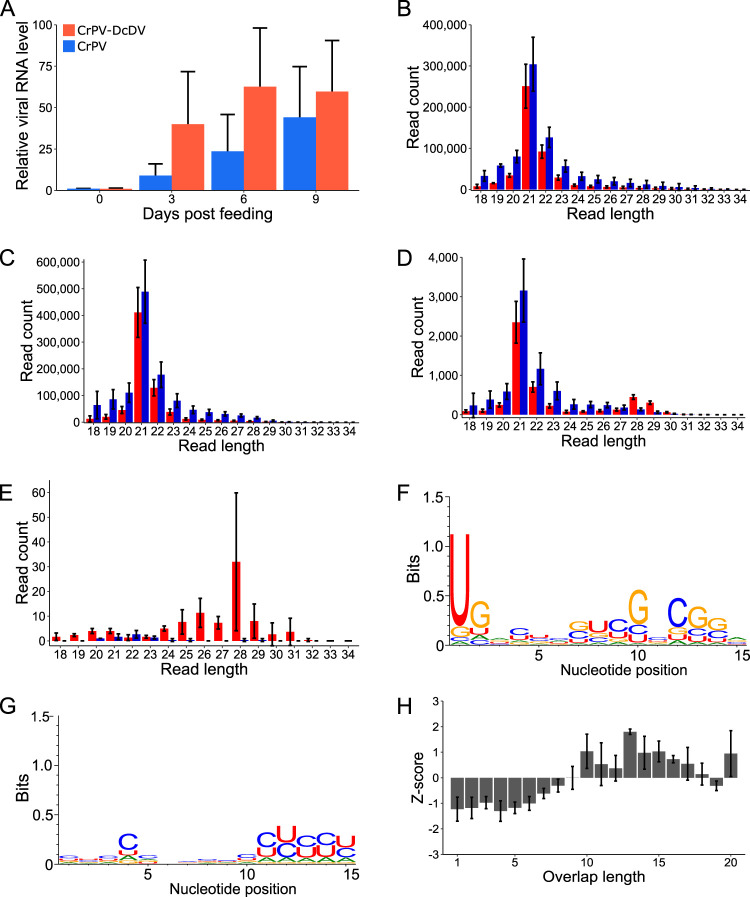
CrPV-DcDV is not targeted by piRNAs during infection initiated by oral acquisition of purified virions. (A) Relative viral RNA levels during infection with wild-type CrPV or CrPV-DcDV. Infection was initiated in CRF-CA *D. citri* by oral acquisition. Insects were allowed to feed for 96 h on a sucrose solution containing 10^9^ TCID_50_ units/ml of wild-type CrPV or CrPV-DcDV. Following the feeding period, insects were moved to *C. macrophylla* plants (day 0 post feeding). Viral RNA levels were assessed by RT-qPCR and normalized based on the expression of actin. The amount of viral RNA present on day 0 was set as 1, and viral RNA levels are shown relative to this value. Bars represent the average viral RNA level in seven pools of three insects. Error bars indicate standard errors of the means. (B to F) Analysis of sRNA sequencing data of sRNAs purified from CRF-CA *D. citri* infected with wild-type CrPV or CrPV-DcDV by oral acquisition as described for panel A. sRNA was purified from pools of 25 *D. citri* collected 9 days post feeding. (B) Length distribution of sRNAs from wild-type CrPV-infected *D. citri* mapped to the wild-type CrPV genome. Red = antisense, blue = sense. Read counts represent averages of results from three independent libraries. Error bars indicate standard deviations. (C) Length distribution of sRNAs from CrPV-DcDV-infected *D. citri* mapped to the CrPV-DcDV genome. Red = antisense, blue = sense. Read counts represent averages of results from three independent libraries. Error bars indicate standard deviations. (D) Length distribution of sRNAs from CrPV-DcDV-infected *D. citri* mapped to the recombinant DcDV sequence present within the CrPV-DcDV genome. Red = antisense, blue = sense. Read counts represent averages of results from three independent libraries. Error bars indicate standard deviations. (E) Length distribution of sRNAs from wild-type CrPV-infected *D. citri* mapped to the recombinant DcDV sequence present within the CrPV-DcDV genome. Red = antisense, blue = sense. Read counts represent averages of results from three independent libraries. Error bars indicate standard deviations. (F and G) Sequence logos for 27-to-32-nt sRNAs from CrPV-DcDV-infected *D. citri* mapped to the recombinant DcDV sequence present within the CrPV-DcDV genome. Sequence logos for antisense sRNAs (F) or sense sRNAs (G) are shown. Data represent results from three pooled libraries. (H) Probability of overlap of the 5′ ends of complementary 27-to-32-nt sRNAs mapping to opposite strands of the recombinant DcDV sequence present within the CrPV-DcDV genome during infection with CrPV-DcDV. Probabilities are shown for the indicated overlap distances and represent averages of results from three independent libraries. Error bars indicate standard deviations. The average Z-score and the standard deviation of the Z-score for an overlap length of 10 nt are shown.

10.1128/mBio.02209-20.8 FIG S8 RT-PCR products produced using primers flanking the site into which the recombinant DcDV sequence was inserted in CrPV-DcDV (primers 15 and 16). RNAs from all biological replicates from each day represented in Fig. 5a (A) or Fig. 6a (B) were pooled and used as the templates for RT-PCR. RNA from CRF-CA *D. citri* was used as a negative control. *In vitro*-transcribed wild-type CrPV or CrPV-DcDV RNA was used as a positive control. DPI = days postinjection. DPF = days postfeeding. Download FIG S8, PDF file, 0.04 MB. Copyright © 2020 Nigg et al. 2020 Nigg et al. This content is distributed under the terms of the Creative Commons Attribution 4.0 International license.

10.1128/mBio.02209-20.9 FIG S9 (A and B) Positions of 21-nt sRNAs mapping to the wild-type CrPV genome (A) or the CrPV-DcDV genome (B) during infection of CRF-CA *D. citri* initiated by intrathoracic injection of virions. Red = antisense sRNAs, blue = sense sRNAs. Read counts represent averages of results from three independent libraries. (C and D) Z-scores for the indicated overlap distances between the 5′ ends of complementary 27-to-32-nt sRNAs mapping to opposite strands of the wild-type CrPV genome (C) or the nonrecombinant portion of the CrPV-DcDV genome (D) during infection of CRF-CA *D. citri* initiated by intrathoracic injection of virions. Z-scores represent averages of results from three independent libraries. Error bars indicate standard deviations. (E and F) Positions of 21-nt sRNAs mapping to the wild-type CrPV genome (E) or the CrPV-DcDV genome (F) during infection of CRF-CA *D. citri* initiated by oral acquisition of virions. Red = antisense sRNAs, blue = sense sRNAs. Read counts represent averages of results from three independent libraries. (G and H) Z-scores for the indicated overlap distances between the 5′ ends of complementary 27-to-32-nt sRNAs mapping to opposite strands of the wild-type CrPV genome (G) or the nonrecombinant portion of the CrPV-DcDV genome (H) during infection of CRF-CA *D. citri* initiated by oral acquisition of virions. Z-scores represent averages of results from three independent libraries. Error bars indicate standard deviations. Download FIG S9, PDF file, 0.3 MB. Copyright © 2020 Nigg et al. 2020 Nigg et al. This content is distributed under the terms of the Creative Commons Attribution 4.0 International license.

To evaluate whether CrPV-DcDV was targeted by ping-pong-dependent piRNAs, we analyzed 27-to-32-nt sRNAs separately depending on whether they mapped to the recombinant DcDV sequence within the CrPV-DcDV genome or to the rest of the CrPV genome. We detected ENS-derived antisense piRNAs mapping to the recombinant DcDV sequence in *D. citri* infected with either wild-type CrPV or CrPV-DcDV (Fig. 5D to F); however, there was no evidence of production of ping-pong-dependent secondary piRNAs from this region during infection with CrPV-DcDV (ping-pong Z-score = 0.28 ± 0.18) (Fig. 5G and H). Ping-pong signatures were also not observed for the nonrecombinant portion of the CrPV-DcDV genome or for wild-type CrPV (Fig. S9C and D). These results indicate that CrPV-DcDV is not targeted by piRNAs during infection initiated by intrathoracic injection in *D. citri* despite the presence of ENS-derived piRNAs identical to the recombinant DcDV sequence.

Because the expression level of ENS-derived piRNAs is highest in the gut (Fig. 2D), we wanted to examine whether a piRNA-based response to CrPV-DcDV would be detectable during infection initiated by oral acquisition. Thus, we orally inoculated *D. citri* insects from CRF-CA with wild-type CrPV or CrPV-DcDV by allowing the insects to feed on a sucrose solution containing 10^9^ TCID_50_ units/ml of wild-type CrPV or CrPV-DcDV. Following the feeding period, the insects were transferred to Citrus macrophylla plants and viral RNA levels were evaluated every 3 days by RT quantitative PCR (RT-qPCR). Viral RNA levels in the insects were nearly identical for wild-type CrPV and CrPV-DcDV immediately following their removal from the virus-containing sucrose solution, and average viral RNA levels increased in the days following transfer of the insects to plants, although there was a substantial amount of variation between biological replicates (Fig. 6A). These results suggest that while both wild-type CrPV and CrPV-DcDV were infectious in *D. citri* following oral acquisition, individual insects displayed a variety of infection outcomes. Similar results have previously been reported for orally acquired infections with other RNA viruses in insects (49). RT-PCR results indicated the recombinant DcDV sequence was retained throughout the course of oral infection with CrPV-DcDV (Fig. S8B). We sequenced sRNAs from insects collected 9 days after transfer of the insects from the virus-containing sucrose solution to plants. We analyzed these data as described above for the infections initiated by injection and found that while both wild-type CrPV and CrPV-DcDV were targeted by siRNAs, neither virus was targeted by ping-pong-dependent vpiRNAs during oral infection (ping-pong Z-score for 27-to-32-nt sRNAs mapping to the recombinant DcDV sequence during CrPV-DcDV infection = 1.04 ± 0.67) (Fig. 6B to H). Together, our results indicate that despite the presence of endogenous primary piRNAs complementary to a portion of the recombinant viral genome, CrPV-DcDV is not targeted by the piRNA pathway in *D. citri* following infection initiated either orally or by intrathoracic injection.

## DISCUSSION

Primary vpiRNAs were first reported for six RNA viruses in a D. melanogaster ovarian somatic sheet (OSS) cell line (50). However, a subsequent study using whole flies found no evidence for the production of primary or secondary vpiRNAs during infection with eight different RNA viruses or a DNA virus in D. melanogaster (14). To date, ping-pong-dependent vpiRNAs have been found in only a small number of mosquito species and cell lines that express an expanded group of Piwi-family Argonaute proteins.

Aedes albopictus densovirus 1 (AalDV-1) was recently shown to be targeted by both siRNAs and ping-pong-dependent vpiRNAs during persistent infection in *Ae. aegypti*-derived Aag2 cells (51). Densoviruses replicate exclusively in the nucleus (52), and, interestingly, analysis of subcellular sRNA fractions of AalDV-1-infected Aag2 cells revealed that while AalDV-1-derived primary piRNAs are found in both the nucleus and cytoplasm, ping-pong-dependent piRNAs targeting AalDV-1 are found only in the cytoplasm (51). Ago3 and Piwi5 are the sole Piwi-family proteins required for the production of vpiRNAs during infection of Aag2 cells with Sindbis virus (9). As these proteins are expressed only in the cytoplasm, that AalDV-1-derived primary piRNAs are present in the nucleus during infection of Aag2 cells suggests that a different pathway may generate primary vpiRNAs from viral RNA in the nucleus (51). Such a pathway would be distinct from the cytoplasmic Ago3/Piwi5 pathway that is responsible for production of vpiRNAs from Sindbis virus and might rely on a piRNA biogenesis factor expressed in the nucleus, such as zucchini endonuclease (51).

How viruses are marked as substrates for piRNA biogenesis in mosquitoes remains unclear. Several previous reports in mosquitoes and other species have speculated that primary piRNAs derived from EVEs may facilitate the targeting of cognate viruses in a manner analogous to the targeting of TEs by the canonical ping-pong cycle (15–17, 21). Theoretically, such a targeting mechanism could facilitate the production of vpiRNAs in species that do not express the expanded group of Piwi-family Argonaute proteins expressed by some mosquito species and mosquito-derived cell lines. The *D. citri* genome contains a DcDV-derived EVE (denoted ENS) with 86% nucleotide identity to the corresponding portion of the DcDV genome. Here, we found that ENS gives rise to DcDV-specific primary piRNAs in geographically distinct *D. citri* populations. However, we found that ENS is not present in all *D. citri* populations and that *D. citri* insects lacking ENS do not produce endogenous DcDV-specific piRNAs. While more-comprehensive analyses are needed, comparing our results with the known geographic distribution of *D. citri* lineages suggests that ENS may be present in lineage B *D. citri* and absent in lineage A *D. citri*.

We previously established a colony of *D. citri* insects harboring DcDV as a persistent infection (CRF-TW) (27), and we found here that insects from this colony do not possess ENS. Sequencing of sRNAs from CRF-TW *D. citri* revealed the production of DcDV-derived ping-pong-dependent vpiRNAs. Similar results were obtained by sequencing sRNAs from DcDV-infected CRF-Uru *D. citri*, another *D. citri* population which lacks ENS and does not produce any endogenous DcDV-specific piRNAs. These were surprising results, as the *D. citri* repertoire of Piwi-family Argonaute proteins is limited to homologs of those expressed in D. melanogaster, which have so far not been associated with the production of vpiRNAs (35, 53). The production of vpiRNAs in *D. citri* seems to display some virus specificity as vpiRNAs were not seen during infection with *Diaphorina citri* reovirus, *Diaphorina citri* picorna-like virus, *Diaphorina citri* bunyavirus, *Diaphorina citri* flavi-like virus, or CrPV. Notably, these are all RNA viruses that replicate in the cytoplasm, but DcDV is a DNA virus that replicates in the nucleus. While the production of ping-pong-dependent DcDV-derived vpiRNAs is noteworthy, we cannot determine whether this process plays an antiviral role on the basis of the present data.

Densoviruses are known to replicate exclusively in the nucleus (52). Given that vpiRNAs were not produced during infection of *D. citri* with positive-strand RNA viruses, a negative-strand RNA virus, or a double-stranded RNA (dsRNA) virus, future analysis should determine whether DcDV RNA is processed into primary vpiRNAs in the nucleus as was suggested previously for AalDV-1 (51). Interestingly, abundant virus-derived sRNAs within the piRNA size range were also observed during infection of Myzus persicae with Myzus persicae densovirus and during infection of Culex pipiens *molestus* with Mosquito densovirus; however, those sRNAs were not analyzed for the presence of piRNA signatures (54, 55).

In addition to ping-pong-dependent vpiRNAs, our results show that DcDV is also targeted by 21-nt siRNAs. RNA interference mediated by siRNAs is the primary antiviral defense mechanism against RNA viruses in insects and requires cleavage of dsRNA substrates by the RNase III enzyme dicer (56). RNA interference mediated by siRNAs is also known to target dsDNA viruses, and a single-stranded DNA (ssDNA) virus was recently shown to be targeted by siRNAs in Aag2 cells (51, 57–60). Previous reports have suggested that overlapping viral transcripts represent the dsRNA substrate that becomes processed into siRNAs during infection with DNA viruses (51, 57–59). Indeed, we previously found that transcriptional readthrough during transcription of the ambisense DcDV genome leads to the production of nearly genome-length complementary transcripts (27). These transcripts may anneal to form the dsRNA required for production of siRNAs and may constitute the sense and antisense precursors required for the ping-pong cycle.

Although our results indicate that EVE-derived piRNAs are not required for the production of ping-pong-dependent DcDV-derived vpiRNAs, we cannot exclude the possibility that EVE-derived piRNAs could serve as an additional pool of piRNAs that could also contribute to priming of the ping-pong cycle. Thus, to investigate whether EVE-derived piRNAs can prime ping-pong amplification during infection with a virus sharing complementary sequence in *D. citri*, we constructed CrPV-DcDV, a recombinant CrPV harboring 57 nt derived from the DcDV genome and corresponding to ENS. Despite the presence of endogenous primary piRNAs derived from ENS perfectly mapping to the recombinant region of CrPV-DcDV, we did not observe induction of the ping-pong cycle during infection with this virus initiated either by oral acquisition or by intrathoracic injection of virions. The lack of ping-pong cycle activation during infection with CrPV-DcDV was unlikely to have been due to an absence of ping-pong activity in the tissues infected by CrPV-DcDV, as we detected abundant TE-derived ping-pong-dependent piRNAs in dissected *D. citri* guts, heads, hemolymph, ovaries, and testes. These results indicate that the presence of EVE-derived primary piRNAs sharing perfect nucleotide identity with an exogenous virus is not in itself sufficient to mark that virus as a target for ping-pong amplification in *D. citri*. We note that our approach has some limitations. Host responses to virus infection are shaped by coevolution, and there is a possibility that CrPV-DcDV was not targeted by ping-pong-dependent piRNAs due to differences in the infection cycles of DcDV (a nuclear replicating DNA virus that infects *D. citri* in nature) and CrPV (a cytoplasmically replicating RNA virus that does not naturally infect *D. citri*). For example, these may include potential differences in the spatial or physical accessibility of viral RNA to piRNA biogenesis factors. Indeed, our results suggest that RNA viruses are not targeted by piRNAs in *D. citri*. Thus, it is possible that even if EVE-derived piRNAs could prime ping-pong amplification during infection with a cognate DNA virus, cognate RNA viruses may not be susceptible to such targeting by the piRNA pathway. Future research aiming to uncover the mechanisms of vpiRNA production during infection with DNA viruses such as DcDV and AalDV-1 will help to resolve these issues.

We previously reported that while DcDV persistently infects CRF-TW *D. citri*, CRF-CA *D. citri* insects are not susceptible to DcDV infection by oral acquisition or intrathoracic injection (27). We report here that CRF-Uru *D. citri* can be persistently infected with DcDV by either of these routes of infection. As discussed above, CRF-TW and CRF-Uru *D. citri* insects are likely to be of lineage A, while CRF-CA *D. citri* is likely to be of lineage B. At present, no genotypic or phenotypic differences between the two lineages have been described except for nucleotide variation within the mitochondrial cytochrome oxidase subunit I gene (36–38). Genetic background is known to contribute to susceptibility to virus infection within different populations of the same species (61). In the case of densoviruses, a single arginine residue at position 188 within a mucin-like glycoprotein expressed in the midgut epithelium of Bombyx mori strains susceptible to infection with Bombyx mori densovirus (BmDV) was substituted by other amino acids in resistant strains (62). Further analysis showed that this mucin-like glycoprotein is the cellular receptor for BmDV in the midgut epithelium and that the arginine residue at position 188 is required for BmDV binding to this protein (62). EVEs have been suggested to play roles in susceptibility to virus infection through a variety of potential mechanisms (16, 63–65). Our results suggest that ENS is present in lineage B *D. citri* but absent in lineage A *D. citri* and that the presence of ENS correlated with resistance to DcDV infection in the populations tested. However, other genetic differences between the two lineages are likely to exist and we cannot assess whether ENS may be involved with resistance to DcDV infection on the basis of the present data. Nevertheless, our data highlight both genetic and phenotypic differences between the two *D. citri* lineages. Other such differences between the lineages and how they may contribute to susceptibility to DcDV infections should be the subject of future investigations.

## MATERIALS AND METHODS

### Maintenance of *D. citri* insects.

*D. citri* insects were reared on *C. macrophylla* plants in mesh cages (BugDorm, Taichung, Taiwan) at 25 ± 2°C using a 14-h/10-h (light/dark) photoperiod and 60% to 70% relative humidity at the CRF of the University of California, Davis (66). *D. citri* insects from Uruguay and Taiwan were imported under USDA APHIS-PPQ permit P526P-17-02906 by shipping adults and nymphs on *C. macrophylla* or Murraya paniculata cuttings, respectively.

### sRNA sequencing and analysis.

For total insect sRNA sequencing of CRF-CA, CRF-TW, and CRF-Uru *D. citri*, total RNA was extracted from groups of 50 adult *D. citri* insects using TRIzol reagent (Invitrogen, Carlsbad, CA) according to the manufacturer’s instructions but omitting the 75% ethanol wash. For CRF-CA *D. citri* infected with wild-type CrPV or CrPV-DcDV by oral acquisition or injection and for the progeny of CRF-Uru *D. citri* infected with DcDV by injection, sRNAs were extracted from groups of 25 adult *D. citri* insects using TRIzol reagent according to the manufacturer’s instructions but omitting the 75% ethanol wash. For organ-specific sRNA sequencing, multiple dissected organs were combined to be processed as one sample as follows: 200 for gut, head, or hemolymph and 100 for ovary or testis. RNA extraction was done from pooled organ samples with a Direct-zol RNA miniprep kit (Zymo Research, Irvine, CA) following the manufacturer’s instructions. Three pooled samples of each organ were used, with each pool representing one of three biological replicates. For all sRNA sequencing, total RNA was sent to Beijing Genomics Institute for library prep with a TruSeq small RNA sample preparation kit (Illumina, San Diego, CA) and sequencing by 50-bp single-end sequencing with a BGISEQ-500 platform. For all sRNA reads, adaptor sequences were removed with Trim Galore version 0.4.4 (67) and the fastxtoolkit was used to remove reads containing bases with a quality score of <20 (68). The remaining reads were mapped to viral genomes, insect genomes, or TEs using BowTie version 1.2.1.1 (69) and the default settings with the following exceptions: –n 1 –l 20. For analysis of only the reads that were perfectly mapped (specifically indicated in the text), the –v 0 option was used instead of the –n 1 –l 20 options. Sequence logos were produced using WebLogo 3 (70). Ping-pong Z-scores were calculated with signature.py (71).

### Analysis of EVEs.

The DcDV-derived EVE was identified in the *D. citri* genome (Diaci2.0, ftp://ftp.citrusgreening.org/genomes/Diaphorina_citri/assembly/DIACI_v2.0/) by BLASTn using the DcDV genome as a query (GenBank accession no. NC_030296). The DcDV-derived EVE was then inspected by aligning the DcDV genome to the relevant portion of the nucleotide sequence of *D. citri* genomic scaffold ScVcwli_3651 using ClustalW (72). Nucleotide identities shared between ENS and DcDV and between EITR and DcDV were calculated as p-distances with complete deletion of gaps from ClustalW alignments using MEGA 7 (73).

### Identification of TEs.

We identified TEs present within the *D. citri* genome (GenBank accession no. GCA_000475195.1) using RepeatMasker version 4.0.6 (74) with the Metazoa library. In addition, to identify TEs lacking homology to previously annotated TEs, we used RepeatModeler version 1.0.8 (75) to produce a *de novo* hidden Markov model for TEs within the *D. citri* genome which was subsequently used as input for a second analysis using RepeatMasker. All TE identifications were performed using TEAnnotator.py as previously described to produce a single-strand-specific .fasta file containing the sequences of all >100-nt TEs identified in the *D. citri* genome (TE annotation provided in Data Set S1, available at https://doi.org/10.7910/DVN/8J57ZA) (39).

### Nucleic acid extraction, reverse transcription, PCR, and Sanger sequencing.

Unless otherwise specified, DNA was extracted from groups of 25 homogenized *D. citri* insects by phenol-chloroform extraction followed by ethanol precipitation. For analysis of ENS transcription, RNA was extracted from groups of 25 homogenized CRF-CA *D. citri* insects using TRIzol reagent according to the manufacturer's instructions. RNA was treated twice with a Qiagen RNase-free DNase set (Qiagen, Hilden, Germany) to remove DNA. cDNA was prepared from 50 ng RNA with SuperScript IV reverse transcriptase (Invitrogen, Carlsbad, CA) by the use of specific primers (see the legend for Fig. 1) according to the manufacturer’s instructions. To assess retention of the insertion in CrPV-DcDV, RNA was extracted from CrPV or CrPV-DcDV virions using TRIzol LS (Invitrogen, Carlsbad, CA) according to the manufacturer’s instructions. cDNA was prepared from 50 ng virion RNA using an Applied Biosystems high-capacity reverse transcription kit (Applied Biosystems, Foster City, CA) and random primers according to the manufacturer’s instructions, and cDNA was diluted 1:10 prior to PCR. Primer sequences are given in Table S1 in the supplemental material, and their use is described in the corresponding figure legends. All PCRs were performed with CloneAmp HiFi PCR premix (TaKaRa Bio, Mountain View, CA) using 25 ng of DNA, 1 μl of cDNA (for determination of ENS transcription), or 3 μl diluted cDNA (for determination of CrPV-DcDV insertion retention). Details of the PCR protocols are available upon request. PCR products were analyzed by 0.8% or 2% agarose gel electrophoresis and visualized under UV after staining with SYBR safe DNA gel stain (Invitrogen, Carlsbad, CA). PCR products were purified with a Zymoclean gel DNA recovery kit (Zymo Research, Irvine, CA) and sequenced by Quintarabio using the Sanger method.

10.1128/mBio.02209-20.10 TABLE S1 Primers used in this study. Download Table S1, PDF file, 0.1 MB. Copyright © 2020 Nigg et al. 2020 Nigg et al. This content is distributed under the terms of the Creative Commons Attribution 4.0 International license.

### DcDV infections and DcDV qPCR.

DcDV virions were partially purified from 0.5 g of CRF-TW *D. citri* as previously described (27). The number of DcDV genome copies in the virion preparation was determined by qPCR with primers 23 and 24 (see Table S1) as described previously (27). For infections by intrathoracic injection, the virion preparations were diluted to 8.2 × 10^5^ genome copies/μl in 100 mM Tris-HCl (pH 7.5). Adult CRF-Uru *D. citri* insects were anesthetized on ice for approximately 15 min and intrathoracically injected with approximately 200 nl of the diluted virion preparation by manual injection using a syringe fitted to a 34-gauge stainless steel needle (Hamilton, Reno, Nevada). Following injection, insects were maintained on *C. macrophylla* plants. For infections by oral acquisition, the virion preparations were diluted to 8.2 × 10^5^ genome copies/μl in a mixture containing 15% sucrose, 0.1% green food coloring, and 0.4% yellow food coloring prepared in 100 mM Tris-HCl (pH 7.5). Groups of 30 adult CRF-Uru *D. citri* insects were fed on the virus-containing sucrose solution for 96 h by membrane feeding as described previously (27). Following the feeding period, insects were maintained on *C. macrophylla* plants. Day 0 was designated the day that the insects were transferred from the virus-containing sucrose solution to plants. For both types of infection, adult (>1-day-old) insects were used and neither the age nor the gender of the insects was determined prior to infection.

For both types of infection, five pools of three insects were collected every 2 days and DNA was purified from each pool by phenol-chloroform extraction followed by ethanol precipitation as described previously (27). All insects had been collected or removed from the plants by 17 days post infection. During the period of maintenance on plants, the DcDV-injected and -fed insects laid eggs on the plants. Following removal of the DcDV-injected or -fed insects, the plants were maintained to allow development of the progeny. The progeny were removed on the day of emergence to the adult stage, and DNA was extracted from 10 individual progeny insects from each experiment as described above. A pool of 25 progeny of the DcDV-injected *D. citri* was collected at the same time for sRNA purification. The concentration of DcDV genome copies in the DNA pools or in DNA extracted from individual progeny insects was assessed by qPCR with primers 23 and 24 using 25 ng DNA as described previously (27). Technical triplicates were used for all qPCRs.

### Cloning of CrPV-DcDV.

Complementary lyophilized oligonucleotides containing the DcDV sequence to be inserted flanked upstream by 15 nt corresponding to the 3′ end of the CrPV 1A nucleotide sequence and downstream by 15 nt corresponding to the 5′ end of the CrPV 2B nucleotide sequence were suspended to a concentration of 100 μM in 100 mM potassium acetate–300 μM HEPES (pH 7.5) (oligonucleotide sequences, 5′-TCTAATCCTGGTCCTGCAGACCGTTCACCTTCTCCAGGACCTTCTACTGCATATCGCTATTGTAGCGAGGAAGTGCAATCGCGCCCC and 5′-GGGGCGCGATTGCACTTCCTCGCTACAATAGCGATATGCAGTAGAAGGTCCTGGAGAAGGTGAACGGTCTGCAGGACCAGGATTAGA). To anneal these oligonucleotides, the oligonucleotides were mixed in equimolar ratios, incubated at 94°C for 3 min, and allowed to cool slowly to room temperature to form the duplex oligonucleotide. pCrPV-3 (a gift from Shou-Wei Ding) (76) was linearized by inverse PCR with primers 13 and 14, and the duplex oligonucleotide was ligated to the linearized plasmid in a 2:1 insertion/vector molar ratio using NEBuilder HiFi assembly master mix (New England Biolabs, Ipswich, MA) according to the manufacturer’s instructions. The resulting plasmid (designated pCrPV-1A-DcDV) was transformed into chemically competent Escherichia coli DH5α, and the transformed cells were grown on Luria Bertani (LB) agar plates containing 100 μg/ml ampicillin at 28°C for 16 h. The presence of the insertion was verified by colony PCR with primers 15 and 17 and by Sanger sequencing of colony PCR products. Colonies containing the desired plasmid were used to inoculate 5 ml LB broth containing 100 μg/ml ampicillin, and the cultures were incubated at 28°C for 16 h with shaking at 220 rpm. Plasmids were purified from overnight cultures with a QIAprep Spin miniprep kit (Qiagen, Hilden, Germany) according to the manufacturer’s instructions.

To duplicate the 1A cleavage site on the 3′ end of the recombinant DcDV sequence, pCrPV-1A-DcDV was linearized by inverse PCR with primers 17 and 18 and the linearized plasmid was circularized by blunt end ligation with T4 DNA ligase (New England Biolabs, Ipswich, MA) according to the manufacturer’s instructions to create pCrPV-1A-DcDV-1A. The ligation product was transformed into chemically competent E. coli DH5α, and plasmids were purified as described above.

### Transfection and infection of S2 cells and virion purification.

pCrPV-3 and pCrPV-1A-DcDV-1A were linearized by digestion with BamHI-HF (New England Biolabs, Ipswich, MA). Wild-type and CrPV-DcDV RNA was prepared by *in vitro* transcription of 1 μg linearized plasmid using a mMESSAGE mMACHINE T7 transcription kit (Invitrogen, Carlsbad, CA) according to the manufacturer’s instructions. At 24 h prior to transfection, 5 × 10^6^ S2 cells were seeded in 2 ml Schneider’s Drosophila media (Thermo Fisher, Waltham, MA) supplemented with 10% heat-inactivated fetal bovine serum in 9.6-cm^2^ wells of a tissue culture plate and incubated at 26°C. The following day, cells were transfected with wild-type CrPV or CrPV-DcDV RNA using TransMessenger transfection reagent (Qiagen, Hilden, Germany) according to the manufacturer’s instructions and the transfected cells were incubated at 26°C. After 72 h, 1 ml of transfected cells was transferred to a 50-ml tissue culture flask containing 3 × 10^6^ S2 cells/ml that had been passed 24 h prior in 6 ml Schneider’s Drosophila media supplemented with 10% heat-inactivated fetal bovine serum. After incubation at 26°C for 96 h, virions were purified from the infected S2 cells by centrifugation at 8,000 rpm for 10 min at 4°C using a Beckman GSA rotor (Beckman Coulter, Brea, CA). The supernatant was then collected and centrifuged at 8,000 rpm for 30 min at 4°C using a Beckman GSA rotor. The supernatant was collected and centrifuged through a 15% sucrose cushion (prepared in 10 mM Tris-HCl [pH 7.5]) at 45,000 rpm for 45 min at 11°C in a Beckman 70.1 Ti rotor (Beckman Coulter, Brea, CA). The pellet was resuspended in 10 mM Tris-HCl (pH 7.5) and then filtered through a 0.22-μm-pore-size filter.

The titer of purified CrPV-DcDV or CrPV virions was measured by endpoint dilution. Briefly, 4 × 10^5^ S2 cells were seeded in 500 μl Schneider’s Drosophila media supplemented with 10% heat-inactivated fetal bovine serum in each well of 24-well plates. Cells were incubated for 24 h at 26°C. After 24 h, cells were infected with 10-fold serial dilutions of purified virions in 100-μl volumes. Dilutions over the range of 10^−5^ to 10^−10^ were used, and six individual wells were used for each dilution. After 72 h, cells were examined for the presence of cytopathic effects and the titer of the undiluted virus stock was calculated from these results using the Reed and Muench method.

For infection of S2 cells by the use of purified virions, 5 × 10^6^ S2 cells were seeded in 2 ml Schneider’s Drosophila media supplemented with 10% heat-inactivated fetal bovine serum in 9.6-cm^2^ wells of a tissue culture plate and incubated at 26°C. After 24 h, 100 TCID_50_ units of wild-type CrPV or CrPV-DcDV virions suspended in 100 μl Schneider’s Drosophila media was added to the wells.

### Microscopy.

Wild-type CrPV or CrPV-DcDV virions (see above) were further purified by centrifugation through a 1.2-g/cm^3^ to 1.6-g/cm^3^ cesium chloride density gradient at 50,000 rpm for 4 h at 11°C in a Beckman SW 65 Ti rotor (Beckman Coulter, Brea, CA). Visible “virus bands” representing virions in the gradients were collected using a Hamilton syringe and diluted 1:10 in 10 mM Tris-HCl (pH 7.5). The diluted virions were then centrifuged at 75,000 rpm for 30 min at 11°C in a Beckman TLA 120 rotor (Beckman Coulter, Brea, CA). The pellet was resuspended in 10 mM Tris-HCl (pH 7.5) and negatively stained with uranyl formate. Negatively stained virion samples were observed by transmission electron microscopy using a JEOL 1230 electron microscope (JEOL, Tokyo, Japan) operating at 100 kV. Mock-infected S2 cells and S2 cells infected with wild-type CrPV or CrPV-DcDV were observed by bright-field microscopy using a Leica DM5000B microscope (Leica, Wetzlar, Germany).

### CrPV infections in *D. citri*.

Adult CRF-CA *D. citri* insects were infected with purified CrPV or CrPV-DcDV virions by intrathoracic injection or membrane feeding exactly as described above for DcDV infections. For injections, purified virions were diluted to 5,000 TCID_50_ units/μl and insects were injected with approximately 200 nl each. Seven pools of three insects each were collected on days 0, 3, 6, and 9 for RNA extraction. Three additional pools of 25 insects each were collected on day 9 for sRNA extraction. For membrane feeding, purified virions were diluted to 10^9^ TCID_50_ units/μl. Five pools of three insects each were collected on days 0, 3, and 5 for RNA extraction. Three additional pools of 25 insects each were collected on day 5 for sRNA extraction.

To determine viral RNA levels, RNA was extracted from the pools of three insects using TRIzol reagent according to the manufacturer’s instructions. RNA pellets were resuspended in 25 μl water, cDNA was prepared from 1 μl RNA (approximately 200 to 400 ng RNA) with a high-capacity cDNA reverse transcription kit using random primers according to the manufacturer’s instructions, and the cDNA was diluted 1:10. Wild-type CrPV and CrPV-DcDV RNA levels were determined using 4.5 μl diluted cDNA for qPCRs with SsoAdvanced Universal SYBR green Supermix (Bio-Rad, Hercules, CA) in 10-μl reaction mixtures. Primers 19 and 20 were used for CrPV RNA, and primers 21 and 22 were used for *D. citri* actin. All primers were used at a final concentration of 0.25 nM. The qPCR conditions consisted of initial denaturation at 98°C for 2 min followed by 40 cycles of 95°C for 10 s and 60°C for 20 s.

CrPV RNA levels were normalized based on the expression of *D. citri* actin using the threshold cycle (2^−ΔΔ^*^CT^*) method (77). Technical triplicates were used for all qPCRs. Diluted cDNAs from each day of evaluation were pooled to assess retention of the insertion in CrPV-DcDV as described above.

### Data availability.

All sRNA sequence data generated for this study were deposited to the NCBI SRA database (BioProject accession no. PRJNA629895).
