# Supplementary material for: Endogenous Viral Element-Derived Piwi-Interacting RNAs (piRNAs) Are Not Required for Production of Ping-Pong-Dependent piRNAs from Diaphorina citri Densovirus
Source: mBio. 2020 Sep 29;11(5):e02209-20. doi: 10.1128/mBio.02209-20 (PMC7527727; doi:10.1128/mBio.02209-20)
Supplement: FIG S1 [file mBio.02209-20-sf001.pdf]

Figure S1

```
CRF-CA   AAAGCGATAA GTTGTCGTAT AAATAGTGGT GTTGGTAGCA TTGTAACTTT AGTATGGACT GTTCA [ 65]
Florida  AAAGCGATAA GTTGTCGTAT AAATAGTGGT GTTGGTAGCA TTGTAACTTT AGTATGGACT GTTCA [ 65]
Pakistan AAAGCGATAA GTTGTCGTAT AAATAGTGGT GTTGGTAGCA TTGTAACTTT AGTATGGACT GTTCA [ 65]
Arizona  AAAGCGATAA GTTGTCGTAT AAATAGTGGT GTTGGTAGCA TTGTAACTTT AGTATGGACT GTTCA [ 65]
CRF-HI   AAAGCGATAA GTTGTCGTAT AAATAGTGGT GTTGGTAGCA TTGTAACTTT AGTATGGACT GTTCA [ 65]

CRF-CA   GCAGCGAGAA AATTTGAACG TCTTGATTGC GTGTGTTATT TTTGTCTTTT ACCAAAATTG CCAAC [130]
Florida  GCAGCGAGAA AATTTGAACG TCTTGATTGC GTGTGTTATT TTTGTCTTTT ACCAAAATTG CCAAC [130]
Pakistan GCAGCGAGAA AATTTGAACG TCTTGATTGC GTGTGTTATT TTTGTCTTTT ACCAAAATTG CCAAC [130]
Arizona  GCAGCGAGAA AATTTGAACG TCTTGATTGC GTGTGTTATT TTTGTCTTTT ACCAAAATTG CCAAC [130]
CRF-HI   GCAGCGAGAA AATTTGAACG TCTTGATTGC GTGTGTTATT TTTGTCTTTT ACCAAAATTG CCAAC [130]

CRF-CA   ACCAGTAGTG AATTTAGTTC CGACGAGGAT GAGTTCCGAC GAGGATTCGT GTCACTCGGT GATCG [195]
Florida  ACCAGTAGTG AATTTAGTTC CGACGAGGAT GAGTTCCGAC GAGGATTCGT GTCACTCGGT GATCG [195]
Pakistan ACCAGTAGTG AATTTAGTTC CGACGAGGAT GAGTTCCGAC GAGGATTCGT GTCACTCGGT GATCG [195]
Arizona  ACCAGTAGTG AATTTAGTTC CGACGAGGAT GAGTTCCAAC GAGGATTCGT GTCACTCGGT GATCG [195]
CRF-HI   ACCAGTAGTG AATTTAGTTC CGACGAGGAT GAGTTCCGAC GAGGATTCGT GTCACTCGGT GATCG [195]

CRF-CA   AGGAGGATCC TCTGAGTTCT GCTCCAGCGA GTCCGGCGAC GAGTCAACAT GGTCACAACT TAAGC [260]
Florida  AGGAGGATCC TCTGAGTTCT GCTCCAGCGA GTCCGGCGAC GAGTCAACAT GGTCACAACT TAAGC [260]
Pakistan AGGAGGATCC TCTGAGTTCT GCTCCAGCGA GTCCGGCGAC GAGTCAACAT GGTCACAACT TAAGC [260]
Arizona  AGGAGGATCC TCTGAGTTCT GCTCCAGCGA GTCCGGCGAC GAGTCAACAT GGTCACAACT TAAGC [260]
CRF-HI   AGGAGGATCC TCTGAGTTCT GCTCCAGCGA GTCCGGCGAC GAGTCAACAT GGTCACAACT TAAGC [260]

CRF-CA   GATTCCGAGG AGACGAACTG TACCGTCGTG TTGGCACAGA ATCCCCTGCC CGACACACAA GAAGT [325]
Florida  GATTCCGAGG AGACGAACTG TACCGTCGTG TTGGCACAGA ATCCCCTGCC CGACACACAA GAAGT [325]
Pakistan GATTCCGAGG AGACGAACTG TACCGTCGTG TTGGCACAGA ATCCCCTGCC CGACACACAA GAAGT [325]
Arizona  GATTCCGAGG AGACGAACTG TACCGTCGTG TTGGCACAGA ATCCCCTGCC CGACACACAA GAAGT [325]
CRF-HI   GATTCCGAGG AGACGAACTG TACCGTCGTG TTGGCACAGA ATCCCCTGCC CGACACACAA GAAGT [325]

CRF-CA   CGAATCGATG TTTCAAGAAG ACCGGAGCGC ATCAGTACAT TTCGCAGACG ATCCTCCGAC GGGAG [390]
Florida  CGAATCGATG TTTCAAGAAG ACCGGAGCGC ATCAGTACAT TTCGCAGACG ATCCTCCGAC GGGAG [390]
Pakistan CGAATCGATG TTTCAAGAAG ACCGGAGCGC ATCAGTACAT TTCGCAGACG ATCCTCCGAC GGGAG [390]
Arizona  CGAATCGATG TTTCAAGAAG ACCGGAGCGC ATCAGTACAT TTCGCAGACG ATCCTCCGAC GGGAG [390]
CRF-HI   CGAATCGATG TTTCAAGAAG ACCGGAGCGC ATCAGTACAT TTCGCAGACG ATCCTCCGAC GGGAG [390]

CRF-CA   CCCGGAAGAA GAAATCTATT CTGAAACGGG CGACGAAAAA GAAAACGACT CCCAAAGCGA ACACA [455]
Florida  CCCGGAAGAA GAAATCTATT CTGAAACGGG CGACGAAAAA GAAAACGACT CCCAAAGCGA ACACA [455]
Pakistan CCCGGAAGAA GAAATCTATT CTGAAACGGG CGACGAAAAA GAAAACGACT CCCAAAGCGA ACACA [455]
Arizona  CCCGGAAGAA GAAATCTATT CTGAAACGGG CGACGAAAAA GAAAACGACT CCCAAAGCGA ACACA [455]
CRF-HI   CCCGGAAGAA GAAATCTATT CTGAAACGGG CGACGAAAAA GAAAACGACT CCCAAAGCGA ACACA [455]

CRF-CA   GCGAAGAAGA TGCTCACTCT GACTCCTCAG CAACAGACAT CAGTGTGGGA ACGGCAAACC AACAA [520]
Florida  GCGAAGAAGA TGCTCACTCT GACTCCTCAG CAACAGACAT CAGTGTGGGA ACGGCAAACC AACAA [520]
Pakistan GCGAAGAAGA TGCTCACTCT GACTCCTCAG CAACAGACAT CAGTGTGGGA ACGGCAAACC AACAA [520]
Arizona  GCGAAGAAGA TGCTCACTCT GACTCCT-AG CAACAGACAT CAGTGTGGGA ACGGCAAACC AACAA [520]
CRF-HI   GCGAAGAAGA TGCTCACTCT GACTCCTCAG CAACAGACAT CAGTGTGGGA ACGGCAAACC AACAA [520]

CRF-CA   CTTTATGGCA TTCTTGTTCC TCGTTTGAAC GCCCCTGCAG ACCGTTCACC TTCTCCAGGA CCTTC [585]
Florida  CTTTATGGCA TTCTTGTTCC TCGTTTGAAC GCCCCTGCAG ACCGTTCACC TTCTCCAGGA CCTTC [585]
Pakistan CTTTATGGCA TTCTTGTTCC TCGTTTGAAC GCCCCTGCAG ACCGTTCACC TTCTCCAGGA CCTTC [585]
Arizona  CTTTATGGCA TTCTTGTTCC TCGTTTGAAC GCCCCTGCAG ACCGTTCACC TTCTCCAGGA CCTTC [585]
CRF-HI   CTTTATGGCA TTCTTGTTCC TCGTTTGAAC GCCCCTGCAG ACCGTTCACC TTCTCCAGGA CCTTC [585]

CRF-CA   TACTGCATAT CGCTATCGTG GCGAGGAAAA CCCAAAATAT GGACCTAAAA AA-GCAGTGC ATCCG [650]
Florida  TACTGCATAT CGCTATCGTG GCGAGGAAAA CCCAAAATAT GGACCTAAAA AA-GCAGTGC ATCCG [650]
Pakistan TACTGCATAT CGCTATCGTG GCGAGGAAAA CCCAAAATAT GGACCTAAAA AA-GCAGTGC ATCCG [650]
Arizona  TACTGCATAT CGCTATCGTG GCGAGGAAAA CCCAAAATGT GGACCTAAAA AAACAAGGGA TTCCG [650]
CRF-HI   TACTGCATAT CGCTATCGTG GCGAGGAAAA CCCAAAATGT GGACCTAAAA AAACCATGGC TTCCG [650]

CRF-CA   TTGGGGGGAA GGCTTAGCGG T [671]
Florida  TTGGGGGGAA GGCTTAGCGG T [671]
Pakistan TTGGGGGGAA GGCTTAGCGG T [671]
Arizona  TGGGGGGAA- ---------- - [671]
CRF-HI   TTGGGGGAA- ---------- - [671]
```
