# Supplementary figures and images for: Endogenous Viral Element-Derived Piwi-Interacting RNAs (piRNAs) Are Not Required for Production of Ping-Pong-Dependent piRNAs from Diaphorina citri Densovirus
Source: mBio. 2020 Sep 29;11(5):e02209-20. doi: 10.1128/mBio.02209-20 (PMC7527727; doi:10.1128/mBio.02209-20)

Figure S2

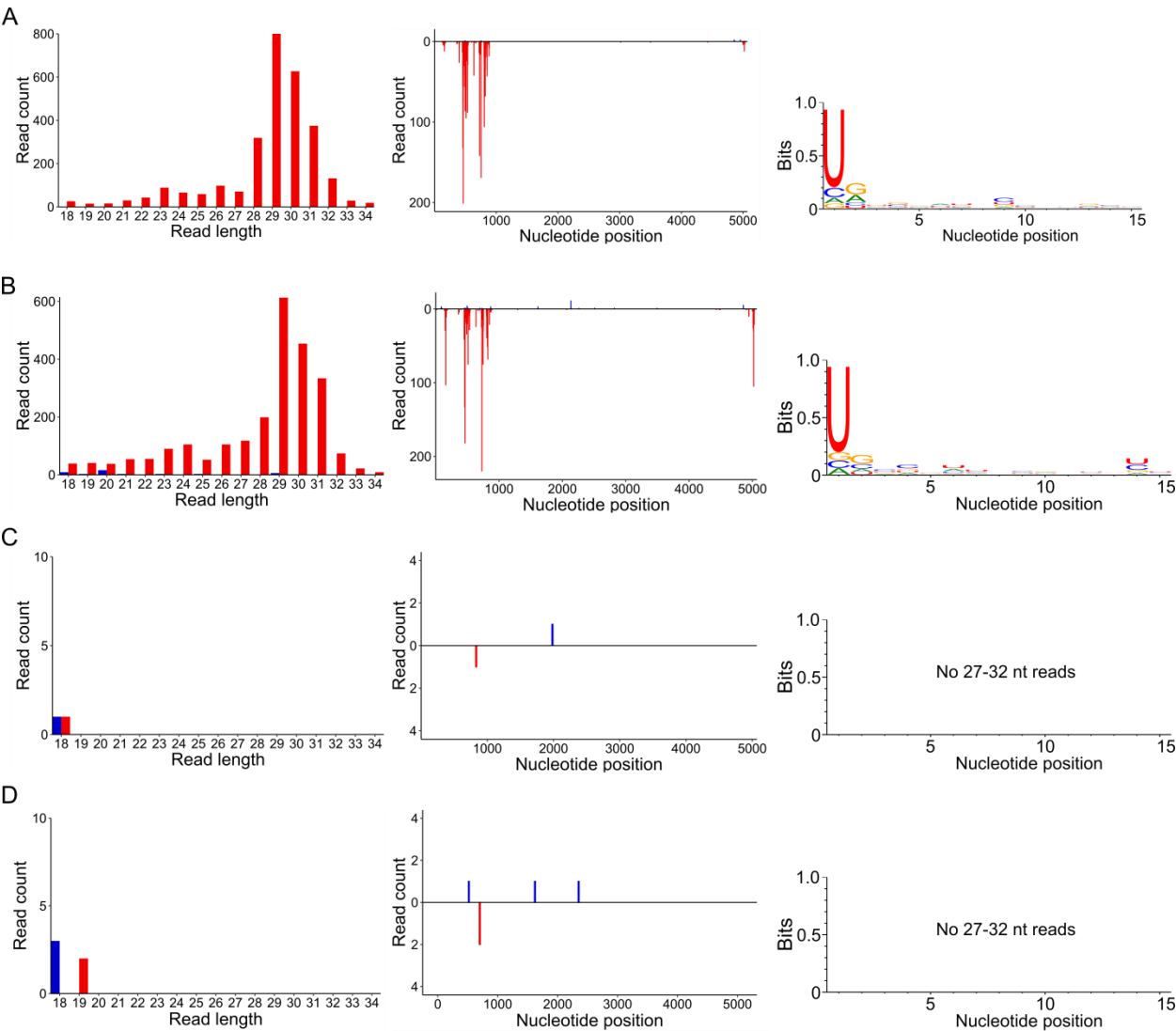

Supplement: FIG S2 [file mBio.02209-20-sf002.pdf]

**Figure S3**

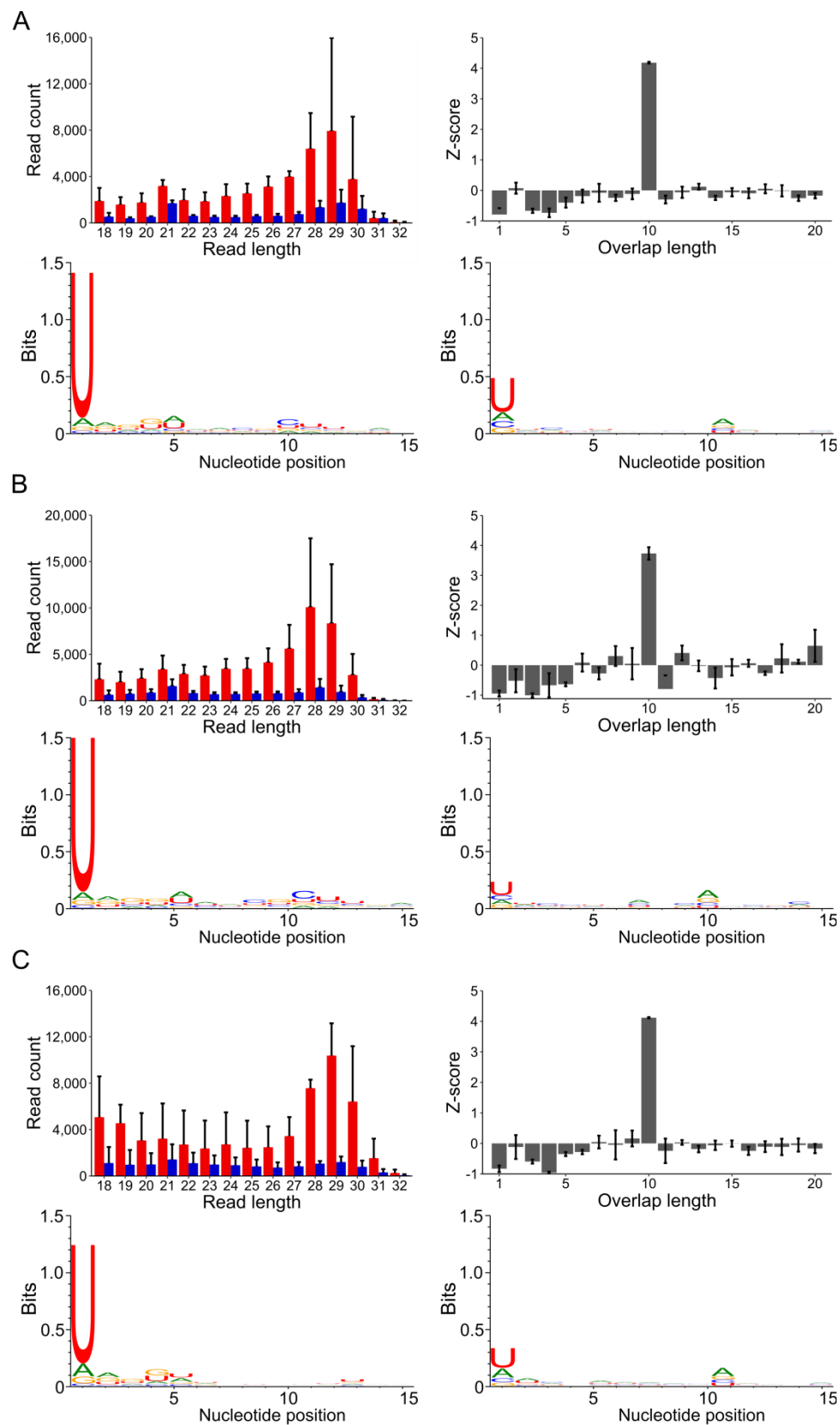

Figure S3 continued

D

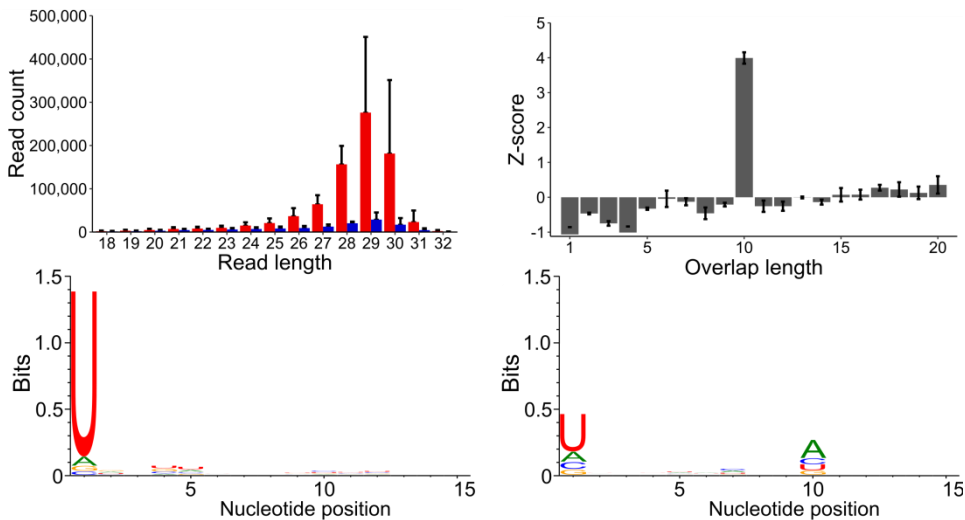

E

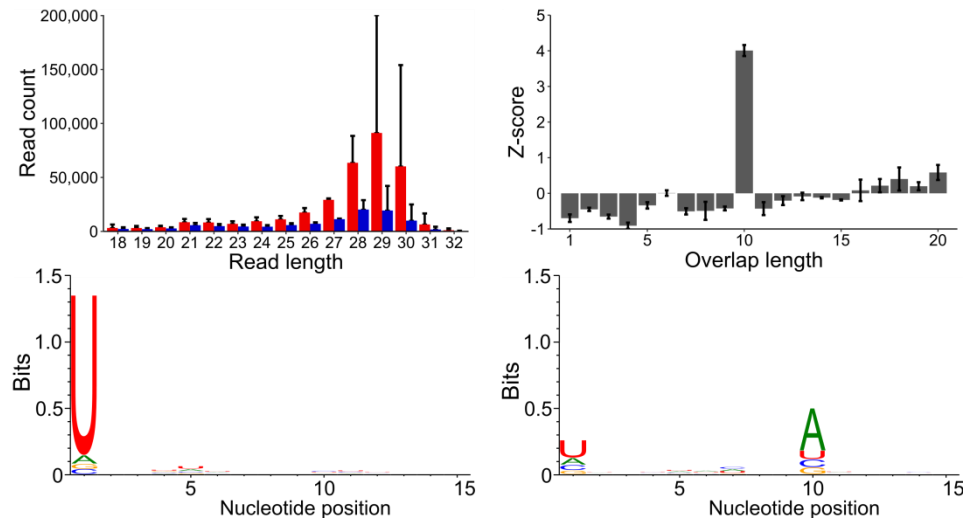

Supplement: FIG S3 [file mBio.02209-20-sf003.pdf]

Figure S4

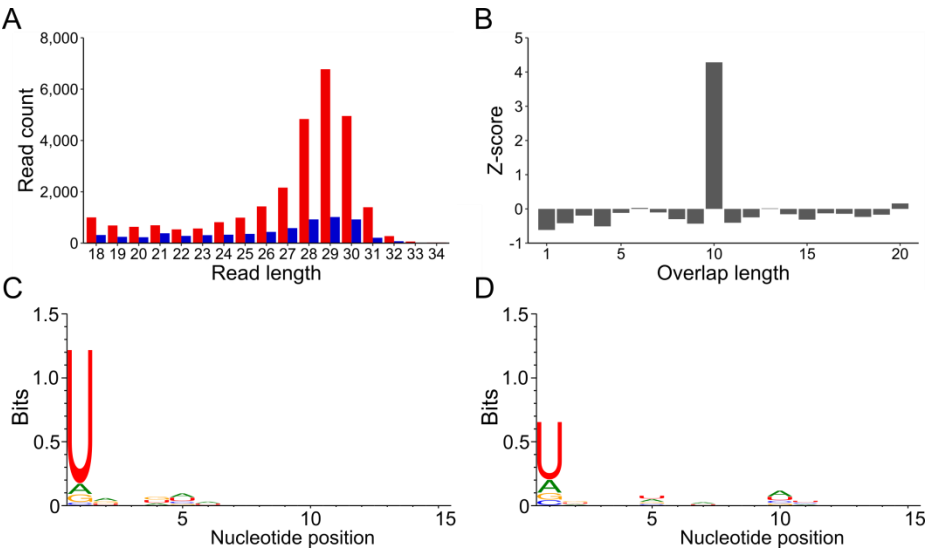

Supplement: FIG S4 [file mBio.02209-20-sf004.pdf]

**Figure S5**

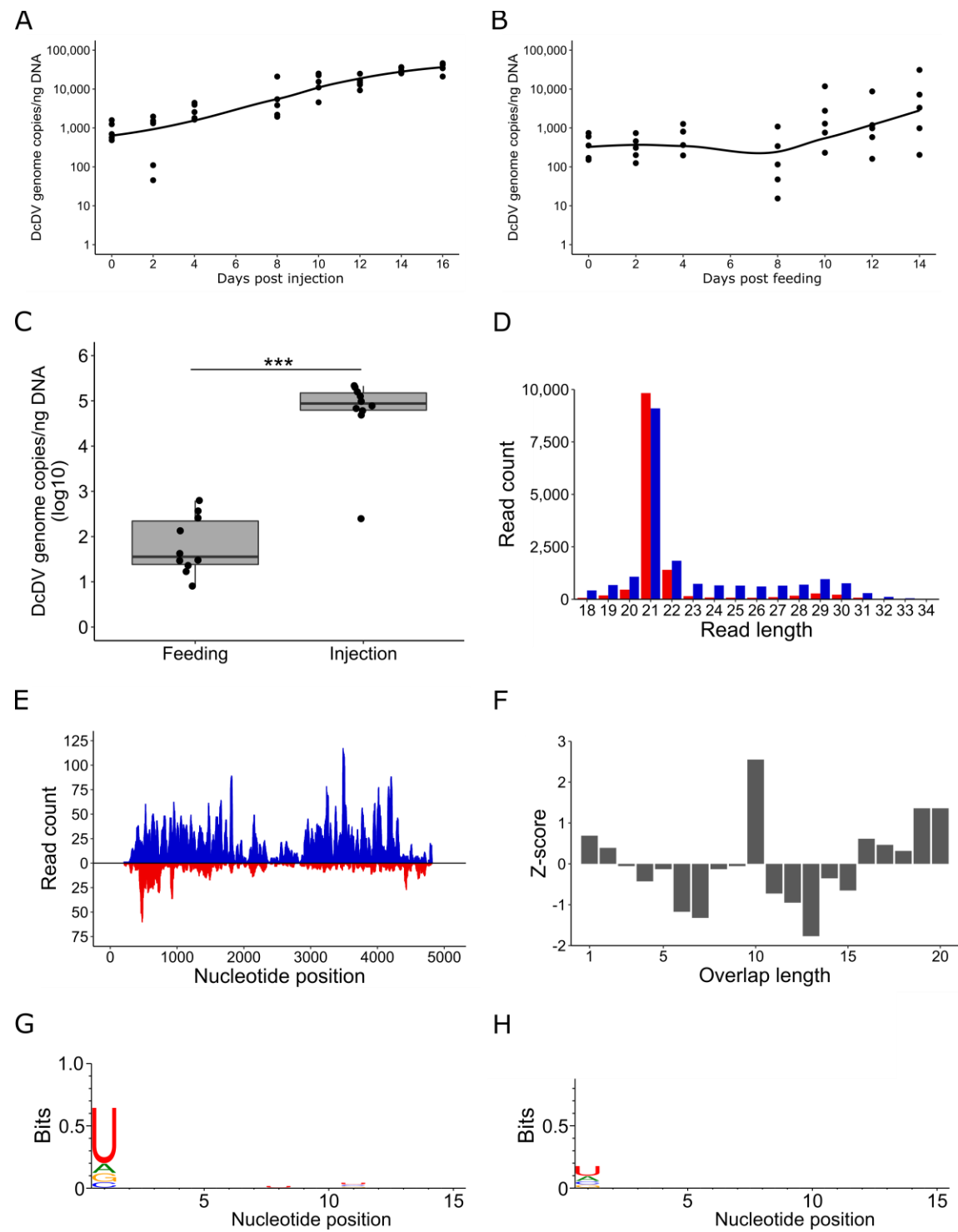

Supplement: FIG S5 [file mBio.02209-20-sf005.pdf]

Figure S6

A

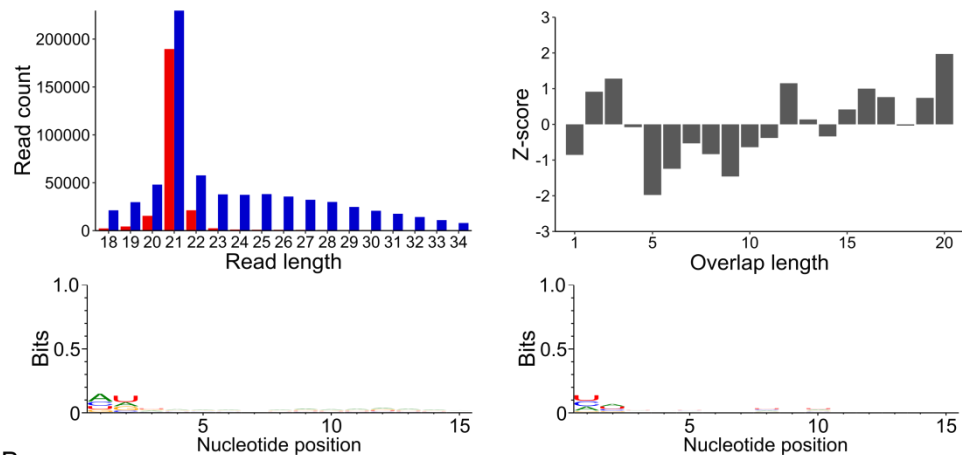

B

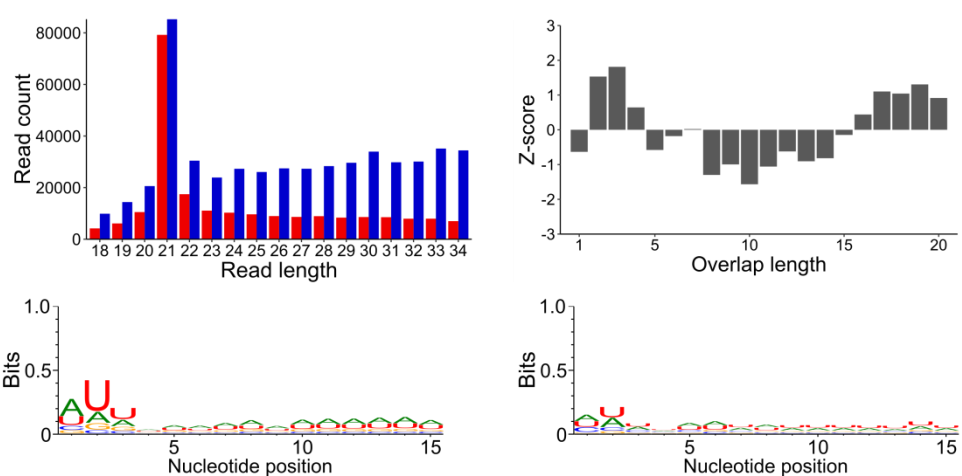

C

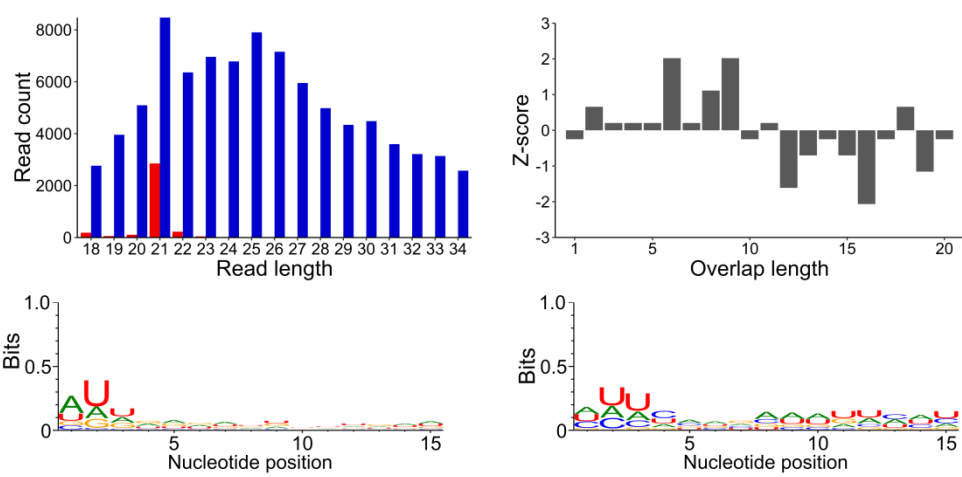

Figure S6 continued

D

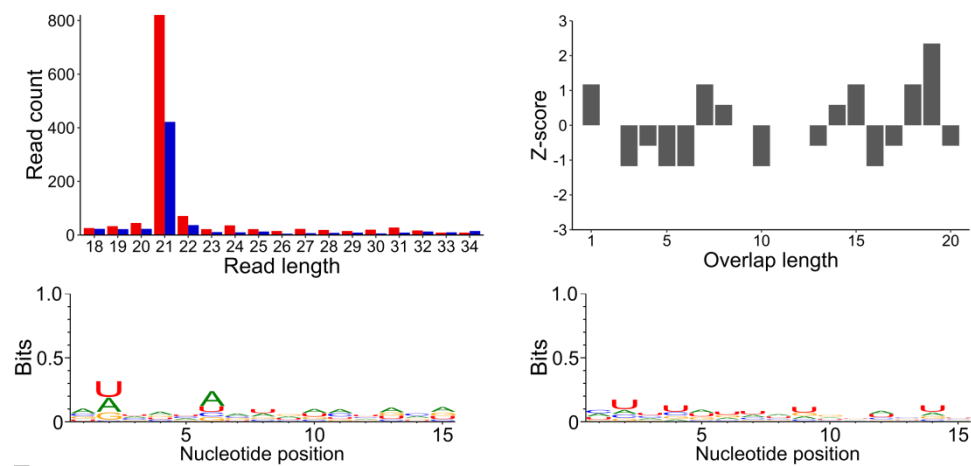

E

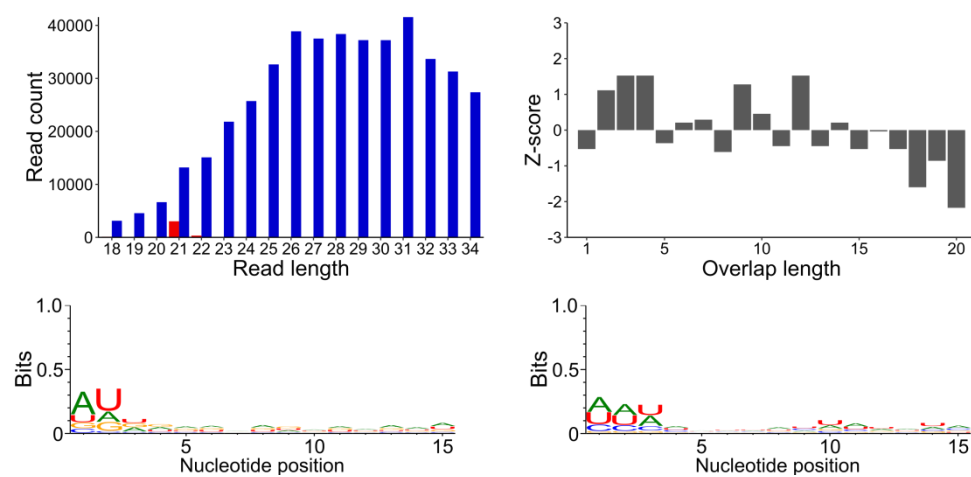

Supplement: FIG S6 [file mBio.02209-20-sf006.pdf]

Figure S8

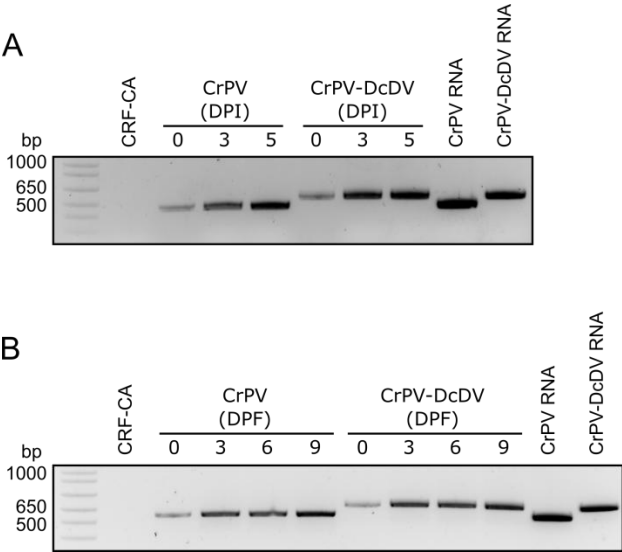

Supplement: FIG S8 [file mBio.02209-20-sf008.pdf]

Figure S9

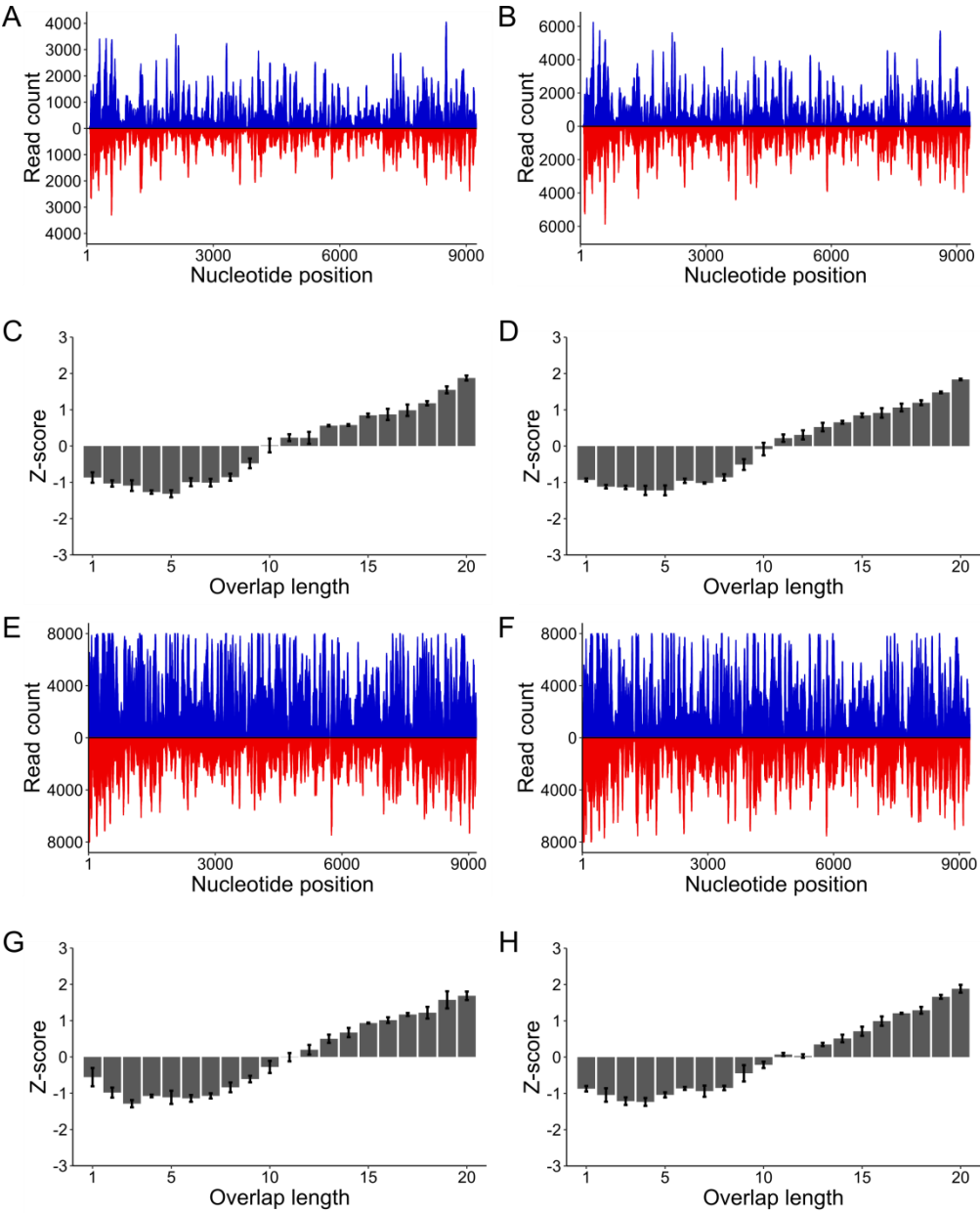

Supplement: FIG S9 [file mBio.02209-20-sf009.pdf]
