# Supplementary material for: Endogenous Viral Element-Derived Piwi-Interacting RNAs (piRNAs) Are Not Required for Production of Ping-Pong-Dependent piRNAs from Diaphorina citri Densovirus
Source: mBio. 2020 Sep 29;11(5):e02209-20. doi: 10.1128/mBio.02209-20 (PMC7527727; doi:10.1128/mBio.02209-20)
Supplement: FIG S7 [file mBio.02209-20-sf007.pdf]

Figure S7

```
CrPV-DcDV (1352-1473)        CTTTTGATGA GTGGTgatgt cgaatctaat cctggtcctG CAGACCGTTC ACCTTCTCCA  [ 60]
DcDV (803-859)               ---------- ---------- ---------- ---------G CAGACCGTTC ACCTTCTCCA  [ 60]
NW_007380266 (8062-8118)     ---------- ---------- ---------- ---------G CAGACCGTTC ACCTTCTCCA  [ 60]
CrPV (1171-1224)             CTTTTGATGA GTGGTgatgt cgaatctaat cctggtcctG ---------- ----------  [ 60]

CrPV-DcDV (1352-1473)        GGACCTTCTA CTGCATATCG CTATTGTAGC GAGGAAgatg tcgaatctaa tcctggtcct  [120]
DcDV (803-859)               GGACCTTCTA CTGCATATCG CTATTGTAGC GAGGAA---- ---------- ----------  [120]
NW_007380266 (8062-8118)     GGACCTTCTA CTGCATATCG CTATCGTGGC GAGGAA---- ---------- ----------  [120]
CrPV (1171-1224)             ---------- ---------- ---------- ---------- ---------- ----------  [120]

CrPV-DcDV (1352-14           GTGCAATCGC GCCCC [135]
DcDV (803-859)               ---------- ----- [135]
NW_007380266 (8062-8118)     ---------- ----- [135]
CrPV (1171-1224)             -TGCAATCGC GCCCC [135]
```
