# Supplementary material for: Endogenous Viral Element-Derived Piwi-Interacting RNAs (piRNAs) Are Not Required for Production of Ping-Pong-Dependent piRNAs from Diaphorina citri Densovirus
Source: mBio. 2020 Sep 29;11(5):e02209-20. doi: 10.1128/mBio.02209-20 (PMC7527727; doi:10.1128/mBio.02209-20)
Supplement: TABLE S1 [file mBio.02209-20-st001.pdf]

Table S1

Primer #	Sequence (5’ → 3’)
1	GGGAAAATCGGTCTCATGCTGCTGTT
2	AAATTGGAGTTAGAAGCTAAAGTTACC
3	TGTAACTTTAGTATGGACTGTTCAGC
4	TTCTTTCACTCCAAACTCTTTCTAGAATGC
5	CCTCTGAGTTCTGCTCCAGC
6	AACAGCAGCATGAGACCGATTTTCCC
7	TGACACCGCTAAGCCTTCC
8	ACACTTTTTTAAAAGCGATAAGTTGTC
9	CCCTGGACTTTGAACAGGAA
10	CATTTGCGGTGAACGATTCC
11	CCAGAACATCGATATGGATCAAC
12	TTGAATTTGGGCTGTTAGTGTC
13	GTGCAATCGCGCCCCGTATATG
14	AGGACCAGGATTAGATTCGACATCAC
15	CCAGAACATCGATATGGATCAAC
16	/phos/TTGAATTTGGGCTGTTAGTGTC†
17	/phos/AGATTCGACATCTTCCTCGCTACAATAGCG†
18	AATCCTGGTCCTGTGCAATCGCGCC
19	GGAAGAATGGCGAATTTTGA
20	CTCGGGAAAGATCCATTTCA
21	TCGTGACATCAAGGAGAAGCTGTGC
22	TTGACCGTCGGGAAGTTCGTAGGAT
23	TGTGTAACCGACAAGCGAAC
24	TCCTCTCGACAACGGAGAAC

† /phos/ indicates 5’ phosphorylation
